# Associations of surgical menopause and hormone replacement therapy with meningioma development

**DOI:** 10.1007/s11060-026-05517-3

**Published:** 2026-03-12

**Authors:** William Zeng, Chloe Jedwood, Ryan Chung, David J. Cote, Jonathan Dallas, Robert G. Briggs, Yetunde B. Omotosho, John Carmichael, Gabriel Zada

**Affiliations:** 1 Keck School of Medicine, University of Southern California, 1975 Zonal Avenue, Los Angeles, CA 90033 USA; 2 Department of Neurosurgery, Keck School of Medicine, University of Southern California, Los Angeles, CA USA; 3 Department of Medicine, Division of Endocrinology, Keck School of Medicine, University of Southern California, Los Angeles, CA USA

**Keywords:** Meningioma, HRT, BSO, Surgical menopause

## Abstract

**Purpose:**

Bilateral salpingo-oophorectomy (BSO) and hormone replacement therapy (HRT) exert opposing effects on systemic sex hormone exposure. Although HRT is frequently prescribed following BSO to mitigate surgical menopause, their combined impact on meningioma risk remains unclear. We evaluated meningioma incidence following BSO, HRT, or both.

**Methods:**

Using the TriNetX database, we identified female patients for the following three categories: BSO only, HRT only, or BSO with subsequent HRT (BSO + HRT). Propensity score matching adjusted for demographics and meningioma risk factors. Outcomes included incident meningioma, cranial and spinal subtypes, time-to-event analyses, and surgical resection rates.

**Results:**

Compared with controls, patients who underwent BSO demonstrated a significantly lower lifetime risk of cranial meningioma (RR:0.85, 95%CI:0.74–0.98, *p* = 0.026). Patients with HRT exposure demonstrated a significantly increased lifetime risk of meningioma diagnosis (RR:1.16, 95%CI:1.09–1.22, *p* < 0.0001). In contrast, patients who underwent BSO + HRT demonstrated a significantly elevated 10-year hazard (HR:1.684, 95%CI:1.40–2.02, *p* < 0.0001) and lifetime risk (RR:1.59, 95%CI:1.33–1.90, *p* < 0.001) of meningioma diagnosis. Risk was highest among BSO + HRT patients with hormonally-driven indications, including uterine fibroids, endometriosis, and gynecologic malignancy (RRs > 2, *p* < 0.001). Among patients who developed meningiomas, those with prior BSO + HRT were significantly less likely to undergo surgical resection (RR:0.41, 95%CI:0.23–0.73, *p* = 0.019) compared with controls.

**Conclusion:**

BSO + HRT patients have an increased risk of meningioma diagnosis. Risks were particularly elevated among women undergoing BSO for hormonally-driven indications. However, there is a lower likelihood of surgical resection among affected patients. These findings have implications for risk stratification, surveillance, and postoperative hormone management.

**Supplementary Information:**

The online version contains supplementary material available at 10.1007/s11060-026-05517-3.

## Introduction

Meningiomas are the most common primary intracranial tumors, accounting for approximately 38% of central nervous system neoplasms with an annual incidence of 6–8 per 100,000 [1, 2]. Although typically benign, meningiomas can cause significant morbidity due to mass effect, subsequent neurological deficits, and need for surgical or radiotherapeutic intervention [2, 3]. Established risk factors for meningioma development include BMI, tobacco use, prior cranial radiation, and endocrine factors, particularly sex hormone signaling [4–7]. Female predominance of meningiomas and frequent expression of progesterone and estrogen receptors support a role for hormonal exposure in tumor development and growth [6, 8, 9]. 

Hormone replacement therapy (HRT) has been associated with an increased incidence of meningioma diagnosis in observational cohorts, especially with long-term use of progestin-containing drugs [10–13]. Endogenous hormonal factors (parity, age at menarche/menopause) have also been proposed as potential modifiers of meningioma risk, although findings have been mixed [2, 10]. The effect of HRT in the context of surgical menopause is not clear. Surgical menopause following bilateral salpingo-oophorectomy (BSO) results in an abrupt withdrawal of estrogen and progesterone, while HRT reintroduces exogenous hormonal exposure [14]. Although HRT is frequently prescribed after BSO to mitigate vasomotor symptoms, osteoporosis, and cardiometabolic effects, the combined influence of BSO and subsequent HRT on meningioma risk has not been well characterized [14, 15]. Existing literature evaluating hormonal influences on meningioma has rarely focused on patients with surgical menopause, and few studies have evaluated whether the indication for BSO, such as endometriosis, fibroids, or gynecologic malignancy, further modifies the risk for meningioma [16, 17]. 

Using a large, multi-institutional national dataset with propensity score matching (PSM), we examined meningioma incidence following BSO with and without HRT and evaluated variation in risk across indications for BSO.

## Methods

### Setting

We conducted a retrospective cohort study using the U.S. Collaborative Network of the TriNetX Research Network (TriNetX, LLC), which aggregates deidentified electronic medical record data from participating healthcare organizations across the U.S., including diagnoses, procedures, medications, and demographics. Diagnoses and procedures are classified using the International Classification of Diseases, Tenth Revision (ICD-10) and Current Procedural Terminology (CPT) manuals, while medications are identified using RxNorm and Anatomical Therapeutic Chemical (ATC) terminology. Because TriNetX provides only aggregated, deidentified, population-level data, this study was exempt from institutional review board approval.

### Cohort

Using CPT and ICD-10 codes, we identified adult female patients (≥ 18 years) who underwent BSO between January 1st, 2005 and November 25th, 2025. TriNetX maps ICD-9-CM and ICD-10-CM codes into unified diagnostic concepts, allowing ICD-10-based queries to capture equivalent ICD-9-coded conditions prior to ICD-10 implementation (2015) [18]. The index event was defined as the earliest date on which a BSO procedure code was recorded. Patients with any diagnosis of meningioma prior to the index event were excluded.

Multiple comparison cohorts were constructed for the evaluation of meningioma incidence. A primary control group consisted of women with no history of BSO and no record of HRT. For our primary analysis, we explored meningioma incidence of patients with BSO and subsequent documented HRT exposure (BSO + HRT), defined by at least one RxNorm-coded systemic estrogen or estrogen-progestin prescription following BSO; patients with BSO alone; and patients with HRT alone. Because contraception is biologically irrelevant following BSO, contraceptive management codes occurring after the BSO were interpreted as proxies for post-surgical hormone management. To evaluate the robustness of our findings and potential sources of bias, we conducted multiple sensitivity analyses, including incorporation of MRI brain exposure into the propensity score model, time-stratified analyses (3- and 5- year follow-up), age-at-HRT initiation stratification as a proxy for exposure duration, exclusion of OCP-coded prescriptions, and comparator surgical cohort analyses (hysterectomy without BSO and cholecystectomy).

To evaluate whether the indication for BSO-modified meningioma risk, the BSO + HRT cohort was stratified by prior diagnoses of endometriosis, uterine fibroids, gynecologic malignancy, or genetic predisposition to breast or ovarian cancer. Each subgroup was compared to patients who had neither BSO nor HRT. For analyses of meningioma treatment, patients developing meningioma after BSO + HRT were compared with matched patients with meningioma and no history of BSO or HRT. All codes are provided in Supplementary Table A1.

### Statistics

All analyses were conducted within the TriNetX platform, with the index date defined as the date of BSO for exposed cohorts. PSM was performed using age, race, and comorbidities associated with meningioma risk, including obese BMI and tobacco use for all analyses [7]. Obese BMI was defined as > 85th percentile, chosen to approximate obesity prevalence (~ 16% in 2022) under the percentile-based constraints of TriNetX [19]. Although prior ionizing radiation is a well-established risk factor for meningioma development, radiation exposure was not included in the primary PSM because in the context of malignancy-related BSO, radiation typically occurs after the indication for surgery and therefore functions as a mediator, not a baseline confounder [3]. Nevertheless, radiation was included in PSM within a secondary sensitivity model for robustness and to verify the directional consistency of our findings. Additional surgical comorbidities were included for PSM in analyses of meningioma treatment and resection. Greedy nearest-neighbor matching with a caliper of 0.1 pooled standard deviations was used, with standardized mean differences less than 0.1 indicating adequate balance. The primary outcome for incidence analyses was the diagnosis of any meningioma, intracranial meningioma, and spinal meningioma, identified using ICD-10 codes. Relative risk ratios with 95% confidence intervals were calculated. Kaplan-Meier survival analysis with log-rank testing and Cox proportional hazards models were used to assess cumulative incidence and hazard ratios. In the meningioma-free survival analysis, the event of interest was a new meningioma diagnosis; thus, diagnosis of meningioma constituted the failure event. For resection analysis, the outcome was defined as any CPT-coded meningioma resection procedure after the index event of a meningioma diagnosis, including spinal resections identified by laminectomy and dural repair codes. Statistical significance was defined a priori as *p* < 0.05.

## Results

### Study population and baseline characteristics

The study cohort comprised 3,302,823 patients, including 65,387 patients in the BSO + HRT exposure group and 3,237,436 control patients. Baseline demographic and clinical characteristics for our primary outcome are presented in Table 1. Additional characteristics are shown in Tables A2-13. Before PSM, patients in the BSO + HRT group were older (mean age:59.4 ± 14.6 vs. 50.6 ± 25.8 years, *p* < 0.001) and differed across all race and ethnicity categories (all *p* < 0.001). After PSM, 64,824 patients were included in each cohort. The matched cohorts were predominantly white (79.5% vs. 78.9%). Despite matching, age differed by 0.4 years (59.3 ± 14.6 vs. 58.9 ± 15.4), a statistically significant but clinically negligible difference (*p* < 0.001). Among patients with available BMI data, mean BMI percentile was lower in the BSO + HRT than in controls before and after matching for BMI ≥ 85th percentile.

**Table 1:** Patient demographics and propensity score-matching for BSO + HRT and control cohorts

Characteristics	Before matching, No. (%)			After matching, No. (%)		
Demographics	BSO + HRT	Control	*p*-value	BSO + HRT	Control	*p*-value
Patients, No.	65,387	3,237,436		64,824	64,824	
Age at Index (Mean +/- SD)	59.4 ± 14.6	50.6 ± 25.8	< 0.0001	59.3 ± 14.6	59.0 ± 15.4	< 0.0001
Not Hispanic or Latino	82.45%	78.96%	< 0.0001	82.32%	81.32%	< 0.0001
White	79.35%	76.51%	< 0.0001	79.45%	79.24%	0.34
Unknown Ethnicity	10.35%	16.76%	< 0.0001	10.43%	10.32%	0.5
Black or African American	10.08%	11.80%	< 0.0001	9.92%	9.75%	0.29
Hispanic or Latino	7.21%	4.29%	< 0.0001	7.25%	8.37%	< 0.0001
Other Race	3.60%	2.79%	< 0.0001	3.62%	3.86%	0.02
Unknown Race	3.22%	5.06%	< 0.0001	3.25%	3.38%	0.2
Unknown Ethnicity	10.35%	16.76%	< 0.0001	10.43%	10.32%	0.5
Asian	2.82%	3.07%	0.0009	2.83%	2.80%	0.67
Native Hawaiin	0.45%	0.37%	0.0029	0.45%	0.46%	0.71
American Indian or Alaska Native	0.48%	0.40%	0.0037	0.48%	0.52%	0.32
BMI Percentile	31.1 ± 12.4	64.1 ± 31.5	< 0.0001	32.5 ± 14.5	47.8 ± 26.4	< 0.0001
BMI ≥ 85th Percentile			< 0.0001			0.893
Tobacco use	5.16%	2.82%	< 0.0001	5.20%	5.37%	0.184

### Meningioma incidence and risk

Patients who underwent BSO without subsequent HRT demonstrated significantly lower lifetime risk of cranial meningioma diagnosis compared with control (0.23% vs. 0.27%, ARD:-0.04%, RR = 0.85, 95%CI:0.74–0.98, *p* = 0.026). The 10-year hazard, however, was not significantly reduced (0.23% vs. 0.27%, ARD:-0.04%, HR = 0.90, 95%CI:0.78–1.03, *p* = 0.137). No significant differences were observed for lifetime spinal meningioma diagnosis risk or 10-year hazard. Similarly, no significant differences were observed in overall meningioma diagnosis risk or 10-year hazard.

Patients with any history of HRT, but no BSO, demonstrated significantly increased lifetime risk for meningioma diagnosis (0.48% vs. 0.30%, ARD:0.18%, RR:1.17, 95%CI:1.11–1.22, *p* < 0.001). Lifetime cranial meningioma diagnosis risk was similarly elevated (0.31% vs. 0.24%, ARD:0.07%, RR:1.16, 95%CI:1.10–1.23, *p* < 0.0001). For spinal meningiomas, there was no increased lifetime risk for meningioma diagnosis. In addition, there was no significant increase in 10-year hazard for overall, cranial, or spinal meningioma.

**Fig. 1:**
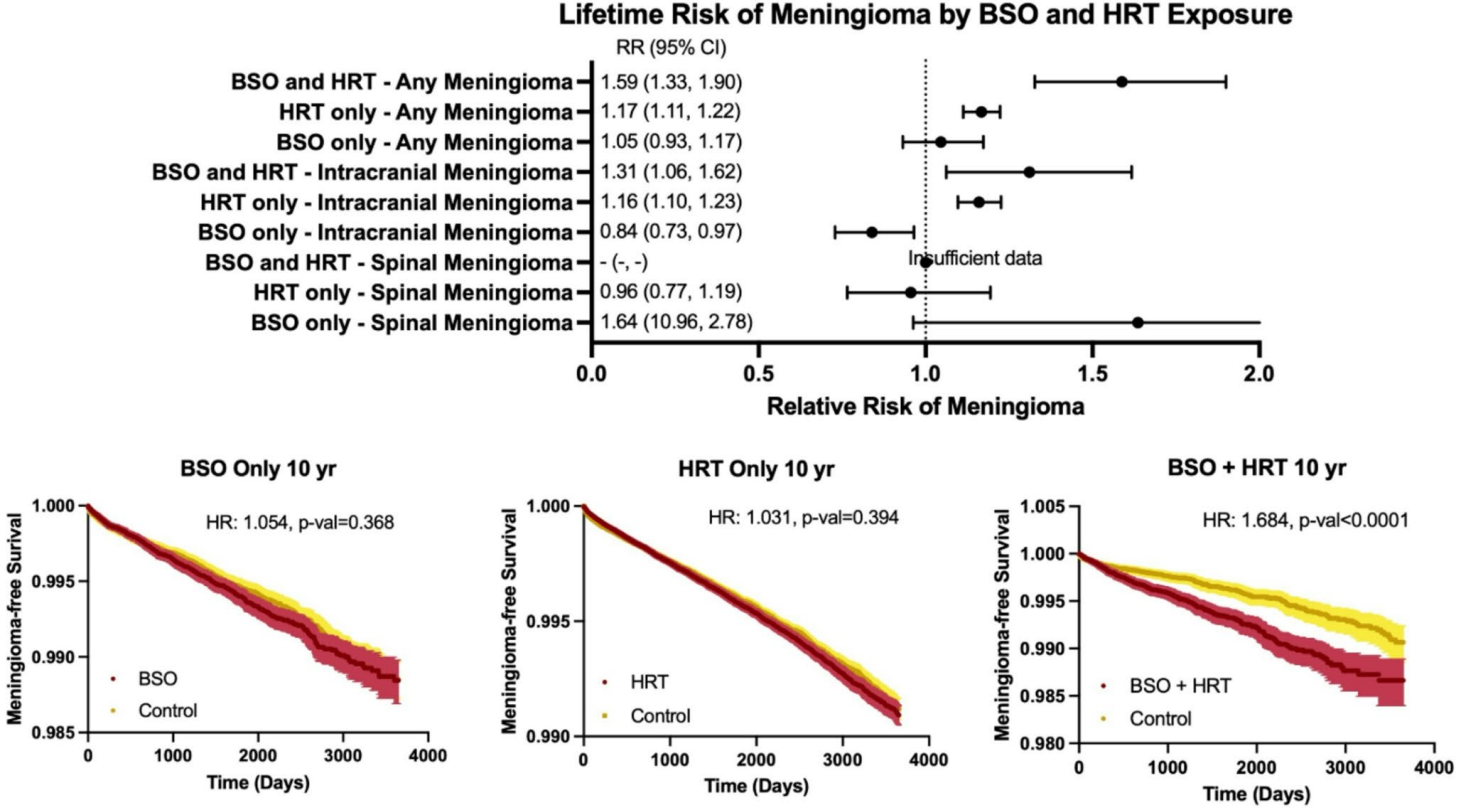
Lifetime risk and 10-year meningioma outcomes by BSO and HRT Exposure. Forest plot (top panel) showing relative risks (points) with 95% confidence intervals (horizontal bars) for meningioma across exposure groups, including BSO, HRT, and BSO + HRT, compared with control. Outcomes are shown for any meningioma, cranial meningioma, and spinal meningioma. The vertical dashed line indicates the null value (RR = 1). Estimates were not reported for groups with insufficient event count. Kaplan-Maier curves (bottom panels) for 10-year meningioma-free survival comparing each exposure group with matched controls. Shaded regions represent 95% confidence intervals. Hazard ratios and corresponding p-values were derived from Cox proportional hazards models

Patients who underwent BSO + HRT demonstrated significantly reduced 10-year meningioma-free survival and elevated meningioma diagnosis hazard over 10 years (98.66% vs. 99.07%, HR:1.68, 95%CI:1.40–2.02, *p* < 0.001). Lifetime diagnosis risk was similarly increased (0.48% vs. 0.30%, ARD:0.18%, RR:1.59, 95%CI:1.33–1.90, *p* < 0.001). This association remained significant in the sensitivity analysis incorporating radiation exposure into the propensity score model (98.65% vs. 99.04%, HR:1.62, 95%CI:1.38–1.94, *p* < 0.001; 0.48% vs. 0.32%, ARD:0.16%, RR:1.52, 95%CI:1.28–1.82, *p* < 0.001). For cranial meningiomas, the associations remained significant (0.31% vs. 0.23%, ARD:0.08%, HR:1.38; 95%CI:1.11–1.70, *p* = 0.003; 0.31% vs. 0.24%, ARD:0.07%, RR:1.31, 95%CI:1.06–1.62, *p* = 0.001), whereas no significant association was observed for spinal meningiomas. Lifetime risk of meningioma diagnosis, cranial meningioma diagnosis, and spinal meningioma diagnosis is summarized in Fig. 1. Kaplan–Meier analysis demonstrated early divergence between groups with curves subsequently running parallel over time, consistent with Cox proportional hazards findings (log-rank χ²=33.64, *p* < 0.001).

**Fig. 2:**
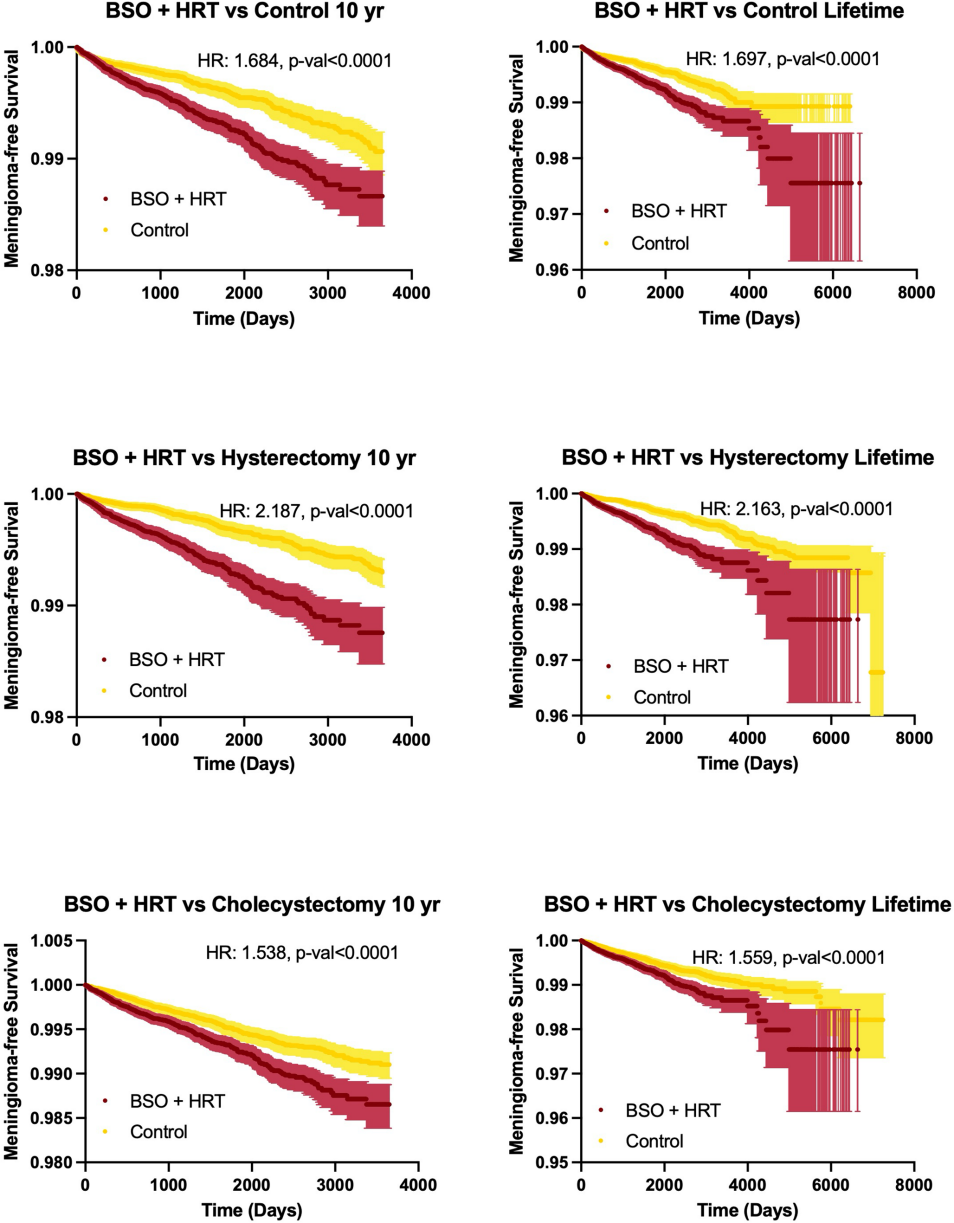
Meningioma-free survival after BSO + HRT compared with control and surgical control cohorts. Kaplan-Meier curves depicting meningioma-free survival among patients undergoing BSO + HRT compared with matched population control (top row). Additional sensitivity analyses using a hysterectomy control group and cholecystectomy control group are also depicted (middle and bottom row respectively). Analyses are shown for both 10-year and lifetime follow-up intervals. Shaded regions represent 95% confidence intervals. Hazard ratios and corresponding p-values were derived from Cox proportional hazards models

When compared with alternative control groups in sensitivity analyses, the BSO + HRT group continued to demonstrate significantly elevated meningioma diagnostic incidence. Compared with hysterectomy controls (excluding BSO), the 10-year hazard and lifetime risk for meningioma diagnosis was significantly increased in the BSO + HRT patients (98.76% vs. 99.30%, HR:2.19, 95%CI:1.77–1.30, *p* < 0.0001; 0.45% vs. 0.34%, ARD:0.11%, RR:2.76, 95%CI:1.08–1.59, *p* < 0.001). Compared with cholecystectomy controls, the same associations persisted (98.65% vs. 99.10%, HR:1.54, 95%CI:1.29–1.83, *p* < 0.001; 0.48% vs. 0.37%, ARD:0.11%, RR:1.31, 95%CI:1.11–1.53, *p* = 0.002). The BSO + HRT group demonstrated lower meningioma-free survival compared to both the hysterectomy (log-rank χ²=56.90, *p* < 0.001) and cholecystectomy control groups (log-rank χ²=26.11, *p* < 0.001). Figure 2 demonstrates the Kaplan-Meier survival curves for the BSO + HRT control and sensitivity comparison groups. Again, the Kaplan-Meier curves visually demonstrated early separation between cohorts across sensitivity analyses, with curves subsequently running parallel over time.

**Fig. 3:**
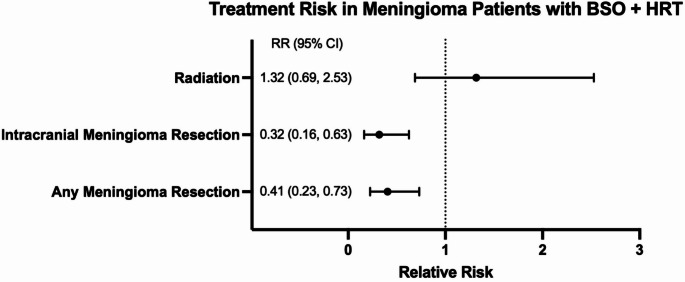
Likelihood of surgical resection among patients with meningioma after BSO + HRT. Forest plot showing relative risks (points) with 95% confidence intervals (horizontal bars) for surgical resection of meningioma in patients with BSO + HRT and confirmed meningioma diagnosis compared with control. The vertical dashed line indicates the null value (RR = 1)

Among patients with recorded diagnosis of meningioma, those with a history of BSO + HRT had significantly lower rates of meningioma resection compared with controls: (1.60% vs. 3.95%, ARD:-2.35%, RR:0.41, 95%CI: 0.23–0.73, *p* = 0.002). Similarly, there were significantly lower rates of cranial meningioma resection (1.16% vs. 3.62%, ARD:-2.46%, RR:0.32, 95%CI:0.16–0.63, *p* = 0.0004). Radiation therapy rates did not differ significantly between groups. Figure 3 summarizes treatments for BSO + HRT patients diagnosed with meningioma.

### Evaluation of clinical indications for BSO

When stratified by indication for BSO + HRT, distinct patterns of meningioma diagnosis risk emerged. Stratification of patients undergoing BSO for malignancy-related reasons revealed multiple subgroups in which BSO + HRT conferred elevated meningioma diagnosis risk. Patients who underwent risk-reducing BSO with subsequent HRT for ER/PR-positive breast cancer demonstrated elevated 10-year hazard (98.71% vs. 99.22%, HR:2.10, 95%CI:1.10–4.01, *p* = 0.0213) and lifetime diagnosis risk (0.76% vs. 0.31%, RR:3.10, 95%CI:1.52–6.32, *p* = 0.007). In contrast, patients who underwent BSO only (without HRT) for ER/PR-positive breast cancer did not demonstrate significantly elevated meningioma diagnosis risk compared with controls. Patients who underwent risk-reducing BSO + HRT for genetic predisposition to malignancy (BRCA1/BRCA2/Lynch) also demonstrated significantly elevated 10-year hazard (98.49% vs. 99.22%, HR:2.78, 95%CI:1.36–5.68, *p* = 0.004) and lifetime risk (0.64% vs. 0.27%, RR:3.21, 95%CI:1.58–6.52, *p* = 0.002). Patients who underwent BSO + HRT after confirmed local malignancy (gynecological, peritoneal) similarly had significantly elevated 10-year hazard (98.42% vs. 99.01%, HR:1.92, 95%CI:1.29–2.88, *p* = 0.001) and lifetime risk (4.21% vs. 1.20%, RR:2.40, 95%CI:1.63–3.54, *p* < 0.001).

Although hysterectomy with ovarian conservation is standard for fibroids and endometriosis, stratified analyses of patients who underwent BSO for these benign indications demonstrated that BSO + HRT was associated with substantially increased meningioma diagnosis [20]. Patients who underwent BSO + HRT for fibroids demonstrated significantly elevated 10-year hazard (98.53% vs. 99.36%, HR:1.84, 95%CI:1.22–2.78, *p* = 0.003) and lifetime diagnostic incidence (0.65% vs. 0.25%, RR:2.22, 95%CI:1.47–3.34, *p* = 0.003) for meningioma. Similarly, patients who underwent BSO + HRT for endometriosis demonstrated a similar pattern of risk (98.41% vs. 99.39%, HR:1.91, 95%CI:1.14–3.19, *p* = 0.012; 0.54% vs. 0.22%, RR:2.34, 95%CI:1.40–3.90, *p* = 0.009).

**Fig. 4:**
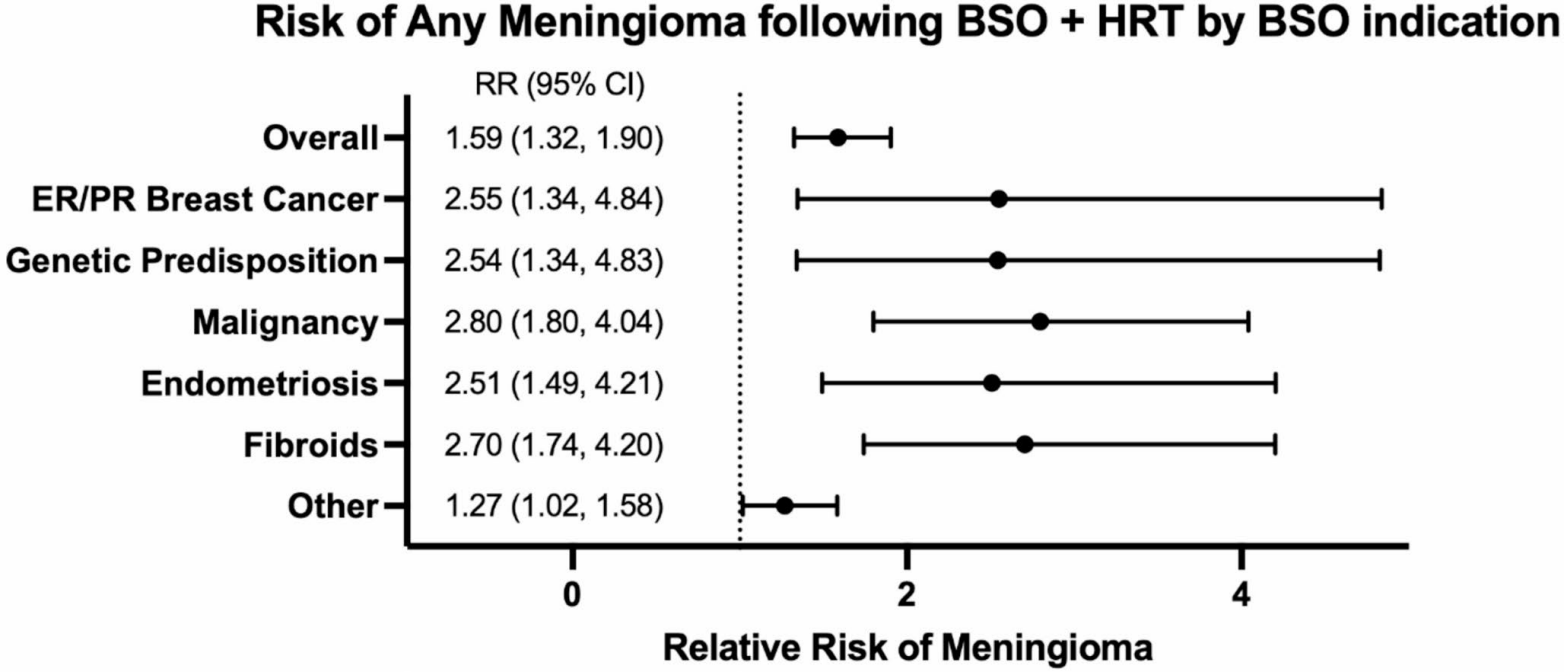
Risk of meningioma following BSO + HRT stratified by indication for BSO. Forest plot showing relative risks (points) with 95% confidence intervals (horizontal bars) for meningioma in patients with BSO + HRT, compared with control. Outcomes are shown for benign, malignant, and unspecified BSO indications. The vertical dashed line indicates the null value (RR = 1)

Patients who underwent BSO + HRT for non-hormonally driven or unspecified indications (after exclusion of hormonally-driven conditions such as malignancy, endometriosis, and fibroids), demonstrated significantly elevated 10-year hazard for meningioma diagnosis (97.29% vs. 98.22%, HR:1.36, 95%CI:1.09–1.69, *p* = 0.007). Lifetime diagnosis risk was similarly elevated (0.48% vs. 0.38%, RR:1.28, 95%CI:1.03–1.60, *p* = 0.006). Figure 4 shows a summary of meningioma risk by BSO + HRT indication. Full incidence, risk, and survival data for all analyses are summarized in Tables A14-27.

### Additional sensitivity analyses

Meningioma incidence in the BSO + HRT cohort at earlier follow-up intervals showed no significant increase at 3 years (99.67% vs. 99.65%, HR:1.13, 95%CI:0.89–1.44, *p* = 0.307) or 5 years (99.32% vs. 99.46%, HR:1.17, 95%CI:0.94–1.44, *p* = 0.156). Among patients initiating HRT prior to age 52 or earlier, the association with meningioma diagnosis remained significant (99.10% vs. 99.51%, HR:1.825, 95%CI:1.15–2.89, *p* = 0.009; 0.32% vs. 0.17%, ARD:0.15%, RR:1.89, 95%CI:1.20–2.99, *p* = 0.006). Among those initiating HRT after 52, the 10-year hazard risk for meningioma diagnosis remained significantly increased ((98.57% vs. 99.02%, HR:1.34, 95%CI:1.08–1.67, *p* = 0.009) while lifetime risk was numerically increased but did not reach statistical significance (0.53% vs. 0.44%, ARD:0.09%, RR:1.21, 95%CI:0.98–1.50, *p* = 0.081). When incorporating previous MRI imaging into the PSM, the 10-year hazard (98.74% vs. 98.97%, HR:1.22, 95%CI:1.01–1.49, *p* = 0.042) and lifetime risk (0.48% vs. 0.34%, ARD:0.14%, RR:1.41, 95%CI:1.15–1.73, *p* < 0.001) for meningioma diagnosis continued to be significantly increased among BSO + HRT patients. When excluding patients with OCP-coded prescriptions, meningioma 10-year hazard (98.74% vs. 99.25%, HR:1.47, 95%CI:1.20–1.81, *p* < 0.001) and lifetime diagnosis risk (0.45% vs. 0.32%, ARD:0.13%, RR:1.41, 95%CI:1.15–1.73, *p* = 0.001) also continued to be significantly increased in BSO + HRT. Full incidence, risk, and survival data for additional sensitivity analyses are summarized in Tables A28-37.

## Discussion

In the present study, we evaluated the 10-year and lifetime diagnosis risk of meningioma among patients who underwent BSO, HRT, or both. BSO alone was associated with reduced lifetime risk for intracranial meningioma diagnosis, whereas HRT alone was associated with increased lifetime diagnosis risk of any meningioma. The lower meningioma incidence observed among patients undergoing BSO was not evident among those who subsequently received HRT. In fact, BSO patients treated with HRT demonstrated a higher observed incidence of meningioma compared with HRT alone, including greater 10-year and lifetime cumulative incidences and a shorter time to diagnosis relative to matched controls. Notably, among patients diagnosed with meningioma, those with prior BSO + HRT were less likely to undergo surgical resection, a finding that may reflect differences in clinical presentation or tumor behavior [21]. Residual differences in BMI were unlikely to explain these findings, as the BSO + HRT group had lower mean BMI percentile values than controls before and after matching for BMI ≥ 85th, which would be expected to bias estimates towards the null. Sensitivity analyses using hysterectomy and cholecystectomy control groups yielded consistent findings. Additional sensitivity analyses were performed to evaluate for surveillance bias, duration-related confounding, and exposure misclassification. These included incorporation of brain MRI exposure into the PSM, time-stratified analyses, age-at-initiation stratification (as a proxy for HRT exposure duration), and exclusion of OCP-coded prescriptions. Across these analyses, effect estimates remained directionally consistent and statistically significant, further supporting that the observed differences between the BSO + HRT and control cohorts likely reflect underlying clinical differences rather than increased diagnostic capture alone.

In supportive analyses stratified by clinical indication for BSO followed by postoperative HRT, fibroids, endometriosis, gynecologic malignancy, and hereditary breast/ovarian cancer predisposition were each associated with a markedly elevated meningioma risk (RR > 2), whereas BSO performed for non-hormone-related or unspecified indications demonstrated more modest elevations in risk (RR ~ 1.2) similar to the unstratified analysis. Our findings warrant cautious interpretation, as causality cannot be inferred from these results.

The observation that BSO alone was associated with a lower lifetime risk of intracranial meningioma diagnosis is consistent with prior literature indicating that removal of endogenous ovarian hormone production may reduce meningioma risk [2, 10]. Reduced lifetime exposure to circulating estrogens and progesterone may decrease proliferative stimulation of hormone receptor-expressing meningeal cells [9]. In our study, the protective association was observed for intracranial but not spinal meningiomas, suggesting possible spatial-anatomical heterogeneity in hormone-sensitivity of meningeal structures.

The role of HRT in meningioma risk remains unclear, but our study contributes to the growing evidence of a positive association. Several prior studies describe conflicting results, with some studies reporting significant meningioma risk elevation with HRT (odds ratios ~ 1.3–1.8) and others reporting null associations [13, 22–25]. Our findings indicate that while history of HRT is associated with a moderately increased lifetime risk of both cranial and spinal meningioma (RR ~ 1.3), there is no associated risk at 3- and 5-year timepoints, suggesting there is a long-term effect of exogenous hormone exposure on meningioma susceptibility.

Rather than exerting an intermediate or attenuated effect, the BSO + HRT group demonstrated a stronger association with meningioma diagnosis (RR ~ 1.6) than HRT alone. This pattern is consistent with effect modification, wherein the association between HRT exposure and meningioma risk differs in the context of prior surgical menopause. Our stratified analyses by BSO indication further supports this interpretation, as the risk between BSO + HRT and incident meningioma diagnosis was stronger for women with other hormone-sensitive conditions. A prior history of fibroids, endometriosis, gynecologic malignancy, or hereditary breast/ovarian cancer predisposition was associated with RRs exceeding two, consistent with literature showing an association between lifetime estrogen and progesterone exposure and meningioma diagnosis [22–25]. A notable contrast emerged among patients undergoing risk-reducing BSO for ER/PR+ breast cancer. Patients who received BSO + HRT demonstrated a markedly elevated meningioma risk (RR > 2.5), whereas those who underwent BSO without HRT did not show a significant increase compared with controls. This divergence suggests that exogenous hormone re-exposure may contribute more substantially to the observed association with meningioma diagnosis than the ER/PR+ breast cancer indication prompting BSO, which alone was not associated with increased risk. In contrast, BSO + HRT performed for non-hormone-related or unspecified indications, most commonly unresolved idiopathic pelvic pain, ovarian cysts, or benign ovarian masses, was associated with more modest risk elevations (RR < 1.3) [16, 17]. Thus, the unstratified BSO + HRT cohort likely includes patients with varying lifetime hormone exposures.

Hormone-sensitive receptor expression in meningiomas offers a clue to how exogenous hormone exposure may influence tumor biology. While mechanistic conclusions cannot be drawn from our data, existing literature on hormone receptors can provide a plausible explanatory framework. Meningiomas frequently express progesterone receptors (PR) and to a lesser extent estrogen receptors (ER), and in vitro data suggest that estrogen and progesterone may stimulate meningioma cell proliferation [6, 9]. A large systematic review reports approximately 78% of meningiomas in women expressed PR, while 15% expressed ER [9]. High PR expression correlates with lower histologic grade and longer time to recurrence, whereas PR-negative or low-PR tumors are more frequently higher grade and associated with worse prognosis [26–30]. ER-positivity, however, is relatively uncommon, and is associated with more aggressive and higher-grade disease, although the clinical significance of ER-positive meningiomas is not as well established in the current literature [31]. These patterns raise the possibility that that HRT may preferentially stimulate the development or enlargement of hormonally-responsive, PR-positive meningiomas. Because lower-grade and indolent PR-positive tumors may not reach a symptomatic threshold requiring operative resection, the net clinical effect would be higher incidence with potentially lower resection rates, consistent with the findings in this study. Given that TriNetX lacks tumor-level hormone receptor data, future studies incorporating ER- and PR-status are needed to evaluate HRT effects more precisely across meningioma subtypes.

Our findings have potential clinical implications as women with prior BSO who receive HRT were observed to have higher relative risks of meningioma diagnosis, particularly when BSO is performed for hormone-sensitive conditions. The combination of higher incidence, earlier onset, and lower resection rates is potentially consistent with the development of smaller, indolent, hormone-responsive tumors, although alternative explanations including residual surveillance bias cannot be excluded. Additionally, because TriNetX lacks granular clinical details on tumor size, location, or competing disease severity, we cannot exclude alternative explanations such as poor surgical candidacy in patients with advanced comorbidities. For neurosurgeons, this suggests that HRT-associated meningiomas may present more often as incidental or asymptomatic lesions, although the absence of imaging and clinical detail within TriNetX limits definitive conclusions. Clinically, our findings support heightening awareness of meningioma as a potential diagnostic consideration in HRT-exposed patients when they present with neurologic symptoms, such as headache, visual changes, seizures, or focal neurologic deficits. The role of continuing or discontinuing HRT after meningioma diagnosis remains an individualized clinical decision and was not directly evaluated in this study. However, emerging evidence suggests that withdrawal of HRT may reverse selected meningioma growth, especially in the spheno-orbital region [32]. These findings may inform discussions among gynecology, endocrinology, and primary care teams when considering systemic hormone therapy after BSO, particularly in individuals with a history of hormone-responsive gynecologic conditions.

### Limitations

This study has several limitations inherent to the use of the TriNetX database. Surveillance bias represents one of the primary limitations of this analysis. Differences in healthcare utilization, imaging frequency and comorbidity burden may influence the likelihood of meningioma detection independent of true biological incidence. Although we performed several additional analyses, including imaging, time, and healthcare utilization-adjusted sensitivity analyses, residual detection bias cannot be fully excluded. While TriNetX provides access to a broad and diverse patient population, the accuracy of diagnoses, procedures, and medication histories is dependent on local documentation and coding practices. Diagnostic coding for meningioma does not reliably distinguish between tumor grade, tumor size, tumor location, or radiographic characteristics, and TriNetX does not capture molecular markers such as PR- or ER-status, which limits our ability to correlate hormonal exposure with receptor-specific tumor biology. Similarly, medication data reflect prescriptions recorded within the contributing health systems. Thus, initiation, discontinuation, or dosing of HRT outside the system may not be fully captured. Moreover, HRT subtype (estrogen-only vs. combined estrogen-progestin) was not stratified, and prescribing patterns may vary by BSO indication. This heterogeneity limits comparability with studies that differentiate estrogen-only versus combined HRT or quantify cumulative dose and duration. Surgical resection as a secondary outcome must also be interpreted cautiously. TriNetX does not provide tumor-level clinical detail such as size, symptomatology or WHO grade, and procedures performed outside participating systems may not be captured. Consequently, lower observed resection rates cannot be assumed to reflect differences in tumor biology or aggressiveness and may instead relate to competing risks, surgical candidacy, or incidental meningioma detection. Despite these limitations, the large sample size, multi-institutional structure, and longitudinal structure of TriNetX offers valuable insights into population-level associations between BSO, HRT exposure, and meningioma risk.

## Conclusions

Although BSO alone is associated with reduced lifetime cranial meningioma diagnosis, BSO + HRT is associated with increased meningioma incidence, but a lower likelihood of surgical intervention among affected patients. Future studies should help to clarify hormonal mechanisms driving these associations and evaluate dose-response effects of specific HRT formulations. Prospective, longitudinal designs with molecular characterization of resected tumors, exposure metrics, and radiographic follow-up can further refine risk stratification and guide screening and surveillance strategies.

## Supplementary Information

Below is the link to the electronic supplementary material.

Supplementary Material 1

## Data Availability

The data that supports the findings of this study are available from the TriNetX Research Network but restrictions apply to their availability. The data used in this study include deidentified individual participant data and associated data dictionaries; however, these data were accessed under license are not publicly available. Detailed cohort definitions, analytic methods, and all diagnostic and medication code lists used to generate the study cohorts are provided in the Supplementary section to facilitate reproducibility for researchers with access to the TriNetX Research Network. All data is available through TriNetX to researchers in accordance with TriNetX data use agreements and policies.
