# Supplementary material for: Associations of surgical menopause and hormone replacement therapy with meningioma development
Source: J Neurooncol. 2026 Mar 12;177(1):49. doi: 10.1007/s11060-026-05517-3 (PMC12982207; doi:10.1007/s11060-026-05517-3)
Supplement: Supplementary file 1 — Supplementary Material 1 [file 11060_2026_5517_MOESM1_ESM.docx]

Table A1: ICD-10-CM, ICD-10-PCS, CPT Codes, and Composite Codes Used for Cohort Definition and Sensitivity Analyses

| **Group** | **Specific Code** | **Description** |
| --- | --- | --- |
| **BSO** |  |  |
| SNOMED | 116144002 | Total abdominal hysterectomy with bilateral salpingo-oophorectomy |
| ICD-10-CM | Z90.722 | Acquired abscence of ovaries, bilateral |
| CPT | 58956 | Bilateral salpingo-oophorectomy with total omentectomy, total abdominal hysterectomy for malignancy |
| CPT | 58953 | Bilateral salpingo-oophorectomy with omentectomy, total abdominal hysterectomy, and radical dissection for debulking |
| CPT | 58954 | Bilateral salpingo-oophorectomy with omentectomy, total abdominal hysterectomy and radical dissection for debulking; with pelvic lymphadenectomy and limited para-aortic lymphadenectomy |
| CPT | 1014216 | Bilateral salpingo-oophorectomy with omentectomy, total abdominal hysterectomy and radical dissection for debulking |
| CPT | 58951 | Resection (initial) of ovarian, tubal or primary peritoneal malignancy with bilateral salpingo-oophorectomy and omentectomy; with total abdominal hysterectomy, pelvic and limited para-aortic lymphadenectomy |
| **HRT** |  |  |
| TNX Curated | 1004 | Hormone Therapy |
| ATC | G03AC | Progestogens |
| ATC | G03AC | HORMONAL CONTRACEPTIVES FOR SYSTEMIC USE |
| ATC | G03C | ESTROGENS |
| ICD-10-CM | Z79.890 | Hormone Replacement Therapy |
| ICD-10-CM | Z79.818 | Long term (current) use of other agents affecting estrogen receptors and estrogen levels |
| ICD-10-CM | Z30.011 | Encounter for initial prescription of contraceptive pills |
| ICD-10-CM | Z30.41 | Encounter for surveillance of contraceptive pills |
| **Hysterectomy** |  |  |
| ICD-10-PCS | 0UT97ZZ | Resection of Uterus, via Natural or Artificial Opening |
| ICD-10-PCS | 0UT94ZZ | Resection of Uterus, Percutaneous Endoscopic Approach |
| ICD-10-PCS | OUT90ZZ | Resection of Uterus, Open Approach |
| *Exclusion: BSO, HRT* | As Above | As Above |
| **Cholecystectomy** |  |  |
| ICD-10-PCS | 0FT40ZZ | Resection of Gallbladder, Open Approach |
| ICD-10-PCS | OFT44ZZ | Resection of Gallbladder, Percutaneous Endoscopic Approach |
| CPT | 47563 | Laparoscopy, surgical; cholecystectomy with cholangiography |
| CPT | 46562 | Laparoscopy, surgical; cholecystectomy with cholangiography |
| CPT | 47600 | Cholecystectomy |
| CPT | 1014153 | Cholecystectomy |
| **Meningioma** |  |  |
| ICD-10-CM | D32 | Benign neoplasm of Meninges |
| ICD-10-CM | D32.0 | Benign neoplasm of cerebral meninges |
| ICD-10-CM | D32.1 | Benign neoplasm of spinal meninges |
| **Cranial Meningioma Resection** |  |  |
| CPT | 61512 | Craniectomy, trephination, bone flap craniotomy; for excision of meninioma, supratentorial |
| CPT | 61519 | Craniectomy for excision of brain tumor, infratentorial or posterior fossa; meningioma |
| **Spinal Meningioma Resection** |  |  |
| CPT | 63276 | Laminectomy for biopsy/excision of intraspinal neoplasm; extradural, thoracic |
| CPT | 63277 | Laminectomy for biopsy/excision of intraspinal neoplasm; extradural, lumbar |
| CPT | 63275 | Laminectomy for biopsy/excision of intraspinal neoplasm; extradural, cervical |
| ICD-10-PCS | 00QT0ZZ | Repair Spinal Meninges, Open Approach |
| ICD-10-PCS | 00QT3ZZ | Repair Spinal Meninges, Percutaneous Approach |
| ICD-10-PCS | 00QT4ZZ | Repair Spinal Meninges, Percutaneous Endoscopic Approach |
| SNOMED | 118886002 | Procedure on spinal meninges |
| **Radiation** |  |  |
| CPT | 77385 | Intensity modulated radiation treatment delivery (IMRT), includes guidance and tracking, when performed; simple |
| CPT | 77386 | Intensity modulated radiation treatment delivery (IMRT), includes guidance and tracking, when performed; complex |
| CPT | 1010904 | Proton beam treatment delivery |
| CPT | 1010895 | Neutron beam treatment delivery |
| ICD-10-PCS | D020JZZ | Stereotactic gamma beam radiosurgery of brain |
| CPT | 63620 | Stereotactic radiosurgery (particle beam, gamma ray, or linear accelerator); 1 spinal lesion |
| ICD-10-PCS | D02 | Stereotactic radiosurgery |
| ICD-10-PCS | DG2 | Stereotactic radiosurgery |
| **Fibroids** |  |  |
| ICD-10-CM | D25 | Leiomyoma of uterus |
| **Endometriosis** |  |  |
| ICD-10-CM | N80 | Endometriosis |
| **Malignancy** |  |  |
| ICD-10-CM | C55 | Malignant neoplasm of uterus, part unspecified |
| ICD-10-CM | C57 | Malignant neoplasm of other and unspecified femal genital organs |
| ICD-10-CM | C56 | Malignant neoplasm of ovary |
| ICD-10-CM | C48.2 | Malignant neoplasm of peritoneum, unspecified |
| **Genetic Predisposition** |  |  |
| ICD-10-CM | Z15.01 | Genetic susceptibility to malignant neoplasm of breast |
| ICD-10-CM | Z15.02 | Genetic susceptibility to malignant neoplasm of ovary |
| ICD-10-CM | Z15.09 | Genetic susceptibility to other malignant neoplasm |
| **ER/PR+ Breast Cancer** |  |  |
| ICD-10-CM | C50 | Malignant neoplasm of breast |
| ICD-10-CM | Z17.0 | Estrogen receptor positive status [ER+] |
| ICD-10-CM | Z17.21 | Progresteron receptor postive status |
| **Non-Hormonal Driven/Unspecified BSO Indication** |  |  |
| *Exclude: Fibroids, Endometriosis, Malignancy, Genetic Predisposition, ER/PR+ Breast Cancer* | As above | As above |

Table A2: Patient Characteristics (HRT vs No BSO/HRT)

| **Characteristics** |  | **Before matching, No. (%)** | |  | **After matching, No. (%)** | |
| --- | --- | --- | --- | --- | --- | --- |
| **Demographics** | **No BSO + HRT** | **Control** | **p-value** | **No BSO + HRT** | **Control** | **p-value** |
| **Patients, No.** | 1,159,298 | 3,237,184 |  | 1,157,445 | 1,157,445 |  |
| **Age at Index (Mean +/- SD)** | 48.9 ± 22.8 | 50.6 ± 25.8 | < 0.0001 | 48.9 ± 22.7 | 49.1 ± 22.7 | < 0.0001 |
| **Not Hispanic or Latino** | 81.72% | 78.96% | < 0.0001 | 81.71% | 81.63% | 0.1111 |
| **White** | 76.95% | 76.51% | < 0.0001 | 76.97% | 76.88% | 0.10 |
| **Unknown Ethnicity** | 13.65% | 16.75% | < 0.0001 | 13.67% | 13.62% | 0.25 |
| **Black or African American** | 13.50% | 11.80% | < 0.0001 | 13.47% | 13.50% | 0.47 |
| **Hispanic or Latino** | 4.63% | 4.29% | < 0.0001 | 4.62% | 4.76% | < 0.0001 |
| **Other Race** | 2.35% | 2.79% | < 0.0001 | 2.36% | 2.35% | 0.76 |
| **Unknown Race** | 3.67% | 5.06% | < 0.0001 | 3.68% | 3.66% | 0.55 |
| **Unknown Ethnicity** | 13.65% | 16.75% | < 0.0001 | 13.67% | 13.62% | 0.25 |
| **Asian** | 2.76% | 3.07% | < 0.0001 | 2.77% | 2.81% | 0.03 |
| **Native Hawaiian** | 0.44% | 0.37% | < 0.0001 | 0.44% | 0.47% | < 0.0001 |
| **American Indian or Alaska Native** | 0.33% | 0.40% | < 0.0001 | 0.33% | 0.33% | 0.66 |
| **BMI Percentile** | 70.4 ± 29.2 | 64.1 ± 31.5 | < 0.0001 | 69.8 ± 29.4 | 70.7 ± 29.6 | 0.0183 |
| **BMI ≥ 85th Percentile** |  |  | < 0.0001 |  |  | 0.006 |
| **Tobacco use** | 4.96% | 2.82% | < 0.0001 | 4.85% | 4.91% | 0.0602 |

Table A3: Patient Characteristics (BSO vs No BSO/HRT)

| **Characteristics** |  | **Before matching, No. (%)** | |  | **After matching, No. (%)** | |
| --- | --- | --- | --- | --- | --- | --- |
| **Demographics** | **BSO + No HRT** | **Control** | **p-value** | **BSO + No HRT** | **Control** | **p-value** |
| **Patients, No.** | 162,035 | 3,237,175 |  | 159,734 | 159,734 |  |
| **Age at Index (Mean +/- SD)** | 61.2 ± 13.6 | 50.6 ± 25.8 | < 0.0001 | 61.1 ± 13.6 | 61 ± 14 | 0.0014 |
| **Not Hispanic or Latino** | 80.40% | 78.96% | < 0.0001 | 80.18% | 79.65% | 0.0002 |
| **White** | 74.59% | 76.51% | < 0.0001 | 74.86% | 74.61% | 0.10 |
| **Unknown Ethnicity** | 11.99% | 16.75% | < 0.0001 | 12.15% | 12.02% | 0.23 |
| **Black or African American** | 13.72% | 11.80% | < 0.0001 | 13.38% | 13.43% | 0.68 |
| **Hispanic or Latino** | 7.60% | 4.29% | < 0.0001 | 7.67% | 8.34% | < 0.0001 |
| **Other Race** | 3.60% | 2.79% | < 0.0001 | 3.61% | 3.69% | 0.23 |
| **Unknown Race** | 4.12% | 5.06% | < 0.0001 | 4.18% | 4.27% | 0.17 |
| **Unknown Ethnicity** | 11.99% | 16.75% | < 0.0001 | 12.15% | 12.02% | 0.23 |
| **Asian** | 3.02% | 3.07% | 0.2137 | 3.02% | 2.96% | 0.30 |
| **Native Hawaiian** | 0.44% | 0.37% | < 0.0001 | 0.45% | 0.46% | 0.6921 |
| **American Indian or Alaska Native** | 0.51% | 0.40% | < 0.0001 | 0.51% | 0.59% | 0.00 |
| **BMI Percentile** | 30.2 ± 9.4 | 64.1 ± 31.5 | < 0.0001 | 30.9 ± 11.7 | 47.1 ± 26.1 | < 0.0001 |
| **BMI ≥ 85th Percentile** |  |  | < 0.0001 |  |  | 1.000 |
| **Tobacco use** | 3.99% | 2.82% | < 0.0001 | 4.05% | 4.07% | 0.7742 |

Table A4: Patient Characteristics (BSO + HRT vs Chole Control)

| **Characteristics** |  | **Before matching, No. (%)** | |  | **After matching, No. (%)** | |
| --- | --- | --- | --- | --- | --- | --- |
| **Demographics** | **BSO + HRT** | **Control** | **p-value** | **BSO + HRT** | **Control** | **p-value** |
| **Patients, No.** | 65,387 | 400,183 |  | 65,384 | 65,384 |  |
| **Age at Index (Mean +/- SD)** | 59.4 ± 14.6 | 47.6 ± 18.1 | < 0.0001 | 59.4 ± 14.6 | 59.4 ± 14.6 | 0.8549 |
| **Not Hispanic or Latino** | 82.45% | 65.44% | < 0.0001 | 82.44% | 82.46% | 0.9478 |
| **White** | 79.35% | 69.27% | < 0.0001 | 79.35% | 79.35% | 1.00 |
| **Unknown Ethnicity** | 10.35% | 17.53% | < 0.0001 | 10.35% | 10.36% | 0.91 |
| **Black or African American** | 10.08% | 12.41% | < 0.0001 | 10.08% | 10.05% | 0.85 |
| **Hispanic or Latino** | 82.45% | 65.44% | < 0.0001 | 82.44% | 82.46% | 0.9478 |
| **Other Race** | 3.60% | 5.96% | < 0.0001 | 3.60% | 3.64% | 0.73 |
| **Unknown Race** | 3.22% | 6.86% | < 0.0001 | 3.22% | 3.22% | 0.94 |
| **Unknown Ethnicity** | 10.35% | 17.53% | < 0.0001 | 10.35% | 10.36% | 0.91 |
| **Asian** | 2.82% | 3.81% | < 0.0001 | 2.82% | 2.80% | 0.84 |
| **Native Hawaiian** | 0.45% | 0.95% | < 0.0001 | 0.45% | 0.46% | 0.7729 |
| **American Indian or Alaska Native** | 0.48% | 0.75% | < 0.0001 | 0.48% | 0.49% | 0.75 |
| **BMI Percentile** | 31.1 ± 12.4 | 53.8 ± 31.4 | < 0.0001 | 31.1 ± 12.4 | 31.4 ± 12 | 0.5586 |
| **BMI ≥ 85th Percentile** |  |  | < 0.0001 |  |  | 0.579 |
| **Tobacco use** | 5.16% | 3.00% | < 0.0001 | 5.16% | 5.10% | 0.5984 |

Table A5: Patient Characteristics (BSO + HRT vs Hysterectomy Control)

| **Characteristics** |  | **Before matching, No. (%)** | |  | **After matching, No. (%)** | |
| --- | --- | --- | --- | --- | --- | --- |
| **Demographics** | **BSO + HRT** | **Control** | **p-value** | **BSO + HRT** | **Control** | **p-value** |
| **Patients, No.** | 65,516 | 105,328 |  | 53,090 | 53,090 |  |
| **Age at Index (Mean +/- SD)** | 59.4 ± 14.6 | 50 ± 12.9 | < 0.0001 | 56.3 ± 13.7 | 56.3 ± 13.6 | 0.9334 |
| **Not Hispanic or Latino** | 82.46% | 74.81% | < 0.0001 | 80.22% | 79.73% | 0.0437 |
| **White** | 79.34% | 59.54% | < 0.0001 | 76.13% | 76.59% | 0.08 |
| **Unknown Ethnicity** | 10.32% | 14.61% | < 0.0001 | 11.53% | 12.34% | < 0.0001 |
| **Black or African American** | 10.08% | 21.38% | < 0.0001 | 11.68% | 11.14% | 0.01 |
| **Hispanic or Latino** | 7.22% | 10.59% | < 0.0001 | 8.25% | 7.94% | 0.0602 |
| **Other Race** | 3.62% | 3.50% | 0.209 | 3.79% | 3.65% | 3.65% |
| **Unknown Race** | 3.22% | 6.74% | < 0.0001 | 3.90% | 4.10% | 0.09 |
| **Unknown Ethnicity** | 10.32% | 14.61% | < 0.0001 | 11.53% | 12.34% | < 0.0001 |
| **Asian** | 2.82% | 6.47% | < 0.0001 | 3.40% | 3.41% | 0.89 |
| **Native Hawaiian** | 0.45% | 1.70% | < 0.0001 | 0.55% | 0.54% | 0.7705 |
| **American Indian or Alaska Native** | 0.48% | 0.67% | < 0.0001 | 0.56% | 0.57% | 0.74 |
| **BMI Percentile** | 31.1 ± 12.4 | 33.3 ± 16.1 | 0.014 | 34.5 ± 17.9 | 32.8 ± 15 | 0.2281 |
| **BMI ≥ 85th Percentile** |  |  | < 0.0001 |  |  | 0.317 |
| **Tobacco use** | 5.16% | 2.47% | < 0.0001 | 4.08% | 4.00% | 0.5228 |

Table A6: Patient Characteristics (BSO + HRT Meningioma vs Meningioma Control)

| **Characteristics** |  | **Before matching, No. (%)** | |  | **After matching, No. (%)** | |
| --- | --- | --- | --- | --- | --- | --- |
| **Demographics** | **Meningioma (BSO + HRT)** | **Control** | **p-value** | **Meningioma (BSO + HRT)** | **Control** | **p-value** |
| **Patients, No.** | 1,006 | 133,132 |  | 1,004 | 1,004 |  |
| **Age at Index (Mean +/- SD)** | 67.7 ± 12.6 | 64.2 ± 15 | < 0.0001 | 67.6 ± 12.6 | 67.7 ± 12.8 | 0.9496 |
| **Not Hispanic or Latino** | 86.58% | 73.57% | < 0.0001 | 86.55% | 87.55% | 0.5062 |
| **White** | 80.22% | 72.12% | < 0.0001 | 80.18% | 83.17% | 0.08 |
| **Unknown Ethnicity** | 7.36% | 20.85% | < 0.0001 | 7.37% | 7.27% | 0.93 |
| **Black or African American** | 10.44% | 12.68% | 0.0329 | 10.46% | 9.26% | 0.37 |
| **Hispanic or Latino** | 6.06% | 5.58% | 0.5065 | 6.08% | 5.18% | 0.3835 |
| **Other Race** | 3.68% | 3.79% | 0.8545 | 3.69% | 1.79% | 0.01 |
| **Unknown Race** | 2.49% | 6.85% | < 0.0001 | 2.49% | 2.49% | 1.00 |
| **Unknown Ethnicity** | 7.36% | 20.85% | < 0.0001 | 7.37% | 7.27% | 0.93 |
| **Asian** | 2.49% | 3.66% | 0.0476 | 2.49% | 2.89% | 0.58 |
| **Native Hawaiian** | 0.99% | 0.45% | 0.0094 | 1.00% | 1.00% | 1.00 |
| **American Indian or Alaska Native** | 0.99% | 0.46% | 0.0129 | 1.00% | 1.00% | 1.00 |
| **BMI Percentile** | 30.2 ± 8.98 | 32 ± 14.3 | 0.4561 | 30.2 ± 9.11 | 35.1 ± 12.7 | 0.0798 |
| **BMI ≥ 85th Percentile** |  |  | 0.6834 |  |  | - |
| **Tobacco use** | 4.97% | 1.74% | < 0.0001 | 4.88% | 3.69% | 0.186 |
| **Alcohol use** | 1.89% | 0.48% | < 0.0001 | 1.89% | 1.39% | 0.3801 |
| **Anticoagulants** | 75.05% | 19.67% | < 0.0001 | 75.00% | 75.80% | 0.6785 |
| **Unspecified mental disorder** | 2.29% | 0.35% | < 0.0001 | 2.09% | 1.30% | 0.1664 |
| **Primary hypertension** | 75.55% | 33.33% | < 0.0001 | 75.50% | 76.89% | 0.4632 |
| **Diabetes mellitus** | 38.57% | 13.21% | < 0.0001 | 38.55% | 38.05% | 0.8185 |
| **Atherosclerotic heart disease** | 26.14% | 6.93% | < 0.0001 | 26.00% | 24.50% | 0.441 |
| **Heart failure** | 19.78% | 4.98% | < 0.0001 | 19.62% | 18.23% | 0.4251 |
| **Chronic kidney disease** | 24.06% | 6.09% | < 0.0001 | 23.90% | 22.81% | 0.5618 |
| **Other chronic obstructive pulmonary disease** | 15.41% | 4.97% | < 0.0001 | 15.34% | 14.34% | 0.5302 |
| **Asthma** | 29.62% | 7.73% | < 0.0001 | 29.58% | 28.39% | 0.555 |

A7: Patient Characteristics (Endometriosis (BSO + HRT) vs No BSO/HRT)

| **Characteristics** |  | **Before matching, No. (%)** | |  | **After matching, No. (%)** | |
| --- | --- | --- | --- | --- | --- | --- |
| **Demographics** | **Endometriosis (BSO + HRT)** | **Control** | **p-value** | **Endometriosis (BSO + HRT)** | **Control** | **p-value** |
| **Patients, No.** | 9,535 | 3,237,436 |  | 9,528 | 9,528 |  |
| **Age at Index (Mean +/- SD)** | 49.3 ± 12.4 | 50.6 ± 25.8 | < 0.0001 | 49.3 ± 12.4 | 48.8 ± 12.9 | 0.02 |
| **Not Hispanic or Latino** | 81.98% | 78.96% | < 0.0001 | 81.97% | 81.93% | 0.9399 |
| **White** | 78.36% | 76.51% | < 0.0001 | 78.35% | 78.33% | 0.97 |
| **Unknown Ethnicity** | 9.85% | 16.76% | < 0.0001 | 9.86% | 9.81% | 0.92 |
| **Black or African American** | 10.44% | 11.80% | < 0.0001 | 10.44% | 10.28% | 0.70 |
| **Hispanic or Latino** | 8.17% | 4.29% | < 0.0001 | 8.18% | 8.26% | 0.8329 |
| **Other Race** | 4.03% | 2.79% | < 0.0001 | 4.03% | 4.05% | 0.94 |
| **Unknown Race** | 3.73% | 5.06% | < 0.0001 | 3.74% | 3.73% | 0.97 |
| **Unknown Ethnicity** | 9.85% | 16.76% | < 0.0001 | 9.86% | 9.81% | 0.92 |
| **Asian** | 2.48% | 3.07% | 0.0007 | 2.48% | 2.47% | 0.96 |
| **Native Hawaiian** | 0.40% | 0.37% | 0.7008 | 0.40% | 0.60% | 0.05 |
| **American Indian or Alaska Native** | 0.57% | 0.40% | 0.0128 | 0.57% | 0.56% | 0.92 |
| **BMI Percentile** | 30.5 ± 8.61 | 64.1 ± 31.5 | < 0.0001 | 30.8 ± 8.69 | 43.5 ± 26.6 | < 0.0001 |
| **BMI ≥ 85th Percentile** |  |  | < 0.0001 |  |  | 0.0016 |
| **Tobacco use** | 5.74% | 2.82% | < 0.0001 | 5.74% | 5.79% | 0.8765 |

A8: Patient Characteristics (Malignancy (BSO + HRT) vs No BSO/HRT)

| **Characteristics** |  | **Before matching, No. (%)** | |  | **After matching, No. (%)** | |
| --- | --- | --- | --- | --- | --- | --- |
| **Demographics** | **Malignancy (BSO + HRT)** | **Control** | **p-value** | **Malignancy (BSO + HRT)** | **Control** | **p-value** |
| **Patients, No.** | 10,600 | 3,237,436 |  | 10,586 | 10,586 |  |
| **Age at Index (Mean +/- SD)** | 60.3 ± 14.1 | 50.6 ± 25.8 | < 0.0001 | 60.2 ± 14.1 | 59.9 ± 14.8 | 0.1096 |
| **Not Hispanic or Latino** | 83.54% | 78.96% | < 0.0001 | 83.52% | 82.51% | 0.0502 |
| **White** | 78.16% | 76.51% | < 0.0001 | 78.16% | 78.01% | 0.79 |
| **Unknown Ethnicity** | 8.12% | 16.76% | < 0.0001 | 8.13% | 8.03% | 0.78 |
| **Black or African American** | 8.80% | 11.80% | < 0.0001 | 8.80% | 8.60% | 0.61 |
| **Hispanic or Latino** | 8.34% | 4.29% | < 0.0001 | 8.35% | 9.47% | 0.0044 |
| **Other Race** | 3.97% | 2.79% | < 0.0001 | 3.98% | 4.12% | 0.60 |
| **Unknown Race** | 4.06% | 5.06% | < 0.0001 | 4.06% | 4.12% | 0.84 |
| **Unknown Ethnicity** | 8.12% | 16.76% | < 0.0001 | 8.13% | 8.03% | 0.78 |
| **Asian** | 4.02% | 3.07% | < 0.0001 | 4.02% | 3.97% | 0.86 |
| **Native Hawaiian** | 0.49% | 0.37% | 0.0508 | 0.49% | 0.63% | 0.17 |
| **American Indian or Alaska Native** | 0.50% | 0.40% | 0.1211 | 0.50% | 0.56% | 0.57 |
| **BMI Percentile** | 32.9 ± 16.4 | 64.1 ± 31.5 | < 0.0001 | 33.3 ± 16.9 | 49.3 ± 25.8 | < 0.0001 |
| **BMI ≥ 85th Percentile** |  |  | < 0.0001 |  |  | 1 |
| **Tobacco use** | 3.62% | 2.82% | < 0.0001 | 3.63% | 3.73% | 0.688 |

Table A9: Patient Characteristics (Genetic Predisposition (BSO + HRT) vs No BSO/HRT)

| **Characteristics** |  | **Before matching, No. (%)** | |  | **After matching, No. (%)** | |
| --- | --- | --- | --- | --- | --- | --- |
| **Demographics** | **Genetic Predisposition (BSO + HRT)** | **Control** | **p-value** | **Genetic Predisposition (BSO + HRT)** | **Control** | **p-value** |
| **Patients, No.** | 5,326 | 3,237,436 |  | 5,316 | 5,316 |  |
| **Age at Index (Mean +/- SD)** | 51.7 ± 12.6 | 50.6 ± 25.8 | 0.0022 | 51.7 ± 12.6 | 51.2 ± 13.6 | 0.0436 |
| **Not Hispanic or Latino** | 84.66% | 78.96% | < 0.0001 | 84.73% | 84.50% | 0.747 |
| **White** | 82.67% | 76.51% | < 0.0001 | 82.79% | 81.55% | 0.09 |
| **Unknown Ethnicity** | 8.04% | 16.76% | < 0.0001 | 7.96% | 7.90% | 0.91 |
| **Black or African American** | 5.30% | 11.80% | < 0.0001 | 5.31% | 4.91% | 0.35 |
| **Hispanic or Latino** | 7.30% | 4.29% | < 0.0001 | 7.32% | 7.60% | 0.5798 |
| **Other Race** | 4.41% | 2.79% | < 0.0001 | 4.31% | 4.97% | 0.11 |
| **Unknown Race** | 3.27% | 5.06% | < 0.0001 | 3.25% | 3.25% | 1.00 |
| **Unknown Ethnicity** | 8.04% | 16.76% | < 0.0001 | 7.96% | 7.90% | 0.91 |
| **Asian** | 3.62% | 3.07% | 0.0197 | 3.61% | 3.56% | 0.88 |
| **Native Hawaiian** | 0.24% | 0.37% | 0.1195 | 0.25% | 0.41% | 0.13 |
| **American Indian or Alaska Native** | 0.49% | 0.40% | 0.3350 | 0.49% | 1.35% | < 0.0001 |
| **BMI Percentile** | 28.5 ± 9.73 | 64.1 ± 31.5 | < 0.0001 | 28.8 ± 9.93 | 43.6 ± 25.9 | < 0.0001 |
| **BMI ≥ 85th Percentile** |  |  | 0.0796 |  |  | 1.000 |
| **Tobacco use** | 3.02% | 2.82% | 0.3619 | 3.03% | 3.48% | 0.1896 |

Table A10: Patient Characteristics (ER/PR Breast Ca (BSO + HRT) vs No BSO/HRT)

| **Characteristics** |  | **Before matching, No. (%)** | |  | **After matching, No. (%)** | |
| --- | --- | --- | --- | --- | --- | --- |
| **Demographics** | **ER/PR Breast Ca (BSO + HRT)** | **Control** | **p-value** | **ER/PR Breast Ca (BSO + HRT)** | **Control** | **p-value** |
| **Patients, No.** | 4,539 | 3,237,436 |  | 4,539 | 4,539 |  |
| **Age at Index (Mean +/- SD)** | 58.8 ± 13.6 | 50.6 ± 25.8 | < 0.0001 | 58.8 ± 13.6 | 58.8 ± 13.6 | 0.9736 |
| **Not Hispanic or Latino** | 84.51% | 78.96% | < 0.0001 | 84.51% | 84.42% | 0.9077 |
| **White** | 79.93% | 76.51% | < 0.0001 | 79.93% | 79.91% | 0.98 |
| **Unknown Ethnicity** | 8.97% | 16.76% | < 0.0001 | 8.97% | 9.06% | 0.88 |
| **Black or African American** | 8.48% | 11.80% | < 0.0001 | 8.48% | 8.46% | 0.97 |
| **Hispanic or Latino** | 6.52% | 4.29% | < 0.0001 | 6.52% | 6.52% | 1 |
| **Other Race** | 4.05% | 2.79% | < 0.0001 | 4.05% | 4.03% | 0.96 |
| **Unknown Race** | 2.71% | 5.06% | < 0.0001 | 2.71% | 2.73% | 0.95 |
| **Unknown Ethnicity** | 8.97% | 16.76% | < 0.0001 | 8.97% | 9.06% | 0.88 |
| **Asian** | 3.66% | 3.07% | 0.0224 | 3.66% | 3.70% | 0.91 |
| **Native Hawaiian** | 0.51% | 0.37% | 0.1450 | 0.51% | 0.51% | 1 |
| **American Indian or Alaska Native** | 0.66% | 0.40% | 0.0065 | 0.66% | 0.66% | 1 |
| **BMI Percentile** | 25.6 ± 6.15 | 64.1 ± 31.5 | 0.0001 | 25.6 ± 6.15 | - | - |
| **BMI ≥ 85th Percentile** |  |  | 0.0001 |  |  | - |
| **Tobacco use** | 4.56% | 2.82% | < 0.0001 | 4.56% | 4.58% | 0.9599 |

Table A11: Patient Characteristics (ER/PR Breast Ca (BSO only) vs No BSO/HRT)

| **Characteristics** |  | **Before matching, No. (%)** | |  | **After matching, No. (%)** | |
| --- | --- | --- | --- | --- | --- | --- |
| **Demographics** | **ER/PR Breast Ca (BSO only)** | **Control** | **p-value** | **ER/PR Breast Ca (BSO only)** | **Control** | **p-value** |
| **Patients, No.** | 7,915 | 3,237,191 |  | 7,915 | 7,915 |  |
| **Age at Index (Mean +/- SD)** | 60.1 ± 13.2 | 50.6 ± 25.8 | < 0.0001 | 60.1 ± 13.2 | 60.1 ± 13.2 | 0.8959 |
| **Not Hispanic or Latino** | 82.69% | 78.96% | < 0.0001 | 82.69% | 82.64% | 0.9331 |
| **White** | 80.83% | 76.51% | < 0.0001 | 80.83% | 80.80% | 0.95 |
| **Unknown Ethnicity** | 11.09% | 16.75% | < 0.0001 | 11.09% | 11.09% | 1.00 |
| **Black or African American** | 9.58% | 11.80% | < 0.0001 | 9.58% | 9.54% | 0.94 |
| **Hispanic or Latino** | 6.22% | 4.29% | < 0.0001 | 6.22% | 6.27% | 0.8954 |
| **Other Race** | 2.72% | 2.79% | 0.7058 | 2.72% | 2.74% | 0.92 |
| **Unknown Race** | 2.87% | 5.06% | < 0.0001 | 2.87% | 2.92% | 0.85 |
| **Unknown Ethnicity** | 11.09% | 16.75% | < 0.0001 | 11.09% | 11.09% | 1.00 |
| **Asian** | 3.12% | 3.07% | 0.7997 | 3.12% | 3.11% | 0.96 |
| **Native Hawaiian** | 0.42% | 0.37% | 0.5358 | 0.42% | 0.43% | 0.9026 |
| **American Indian or Alaska Native** | 0.47% | 0.40% | 0.3780 | 0.47% | 0.47% | 1.00 |
| **BMI Percentile** | 32.2 ± 8.13 | 64.1 ± 31.5 | 0.0014 | 32.2 ± 8.13 | 60 ± 27 | 0.0058 |
| **BMI ≥ 85th Percentile** |  |  | < 0.0001 |  |  | 0.002 |
| **Tobacco use** | 5.72% | 2.82% | < 0.0001 | 5.72% | 5.74% | 0.9727 |

Table A12: Patient Characteristics (Other BSO Indication (BSO + HRT) vs No BSO/HRT)

| **Characteristics** |  | **Before matching, No. (%)** | |  | **After matching, No. (%)** | |
| --- | --- | --- | --- | --- | --- | --- |
| **Demographics** | **Non-Hormonal Driven BSO + HRT** | **Control** | **p-value** | **Non-Hormonal Driven BSO + HRT** | **Control** | **p-value** |
| **Patients, No.** | 37,095 | 3,237,191 |  | 36,568 | 36,568 |  |
| **Age at Index (Mean +/- SD)** | 61.6 ± 14.6 | 50.6 ± 25.8 | < 0.0001 | 61.5 ± 14.6 | 61.3 ± 15.1 | 0.0327 |
| **Not Hispanic or Latino** | 82.78% | 78.96% | < 0.0001 | 82.57% | 81.87% | 0.014 |
| **White** | 81.42% | 76.51% | < 0.0001 | 81.51% | 81.54% | 0.91 |
| **Unknown Ethnicity** | 10.61% | 16.75% | < 0.0001 | 10.75% | 10.65% | 0.67 |
| **Black or African American** | 8.88% | 11.80% | < 0.0001 | 8.70% | 8.52% | 0.38 |
| **Hispanic or Latino** | 6.62% | 4.29% | < 0.0001 | 6.68% | 7.47% | < 0.0001 |
| **Other Race** | 3.51% | 2.79% | < 0.0001 | 3.52% | 3.64% | 0.37 |
| **Unknown Race** | 2.78% | 5.06% | < 0.0001 | 2.82% | 2.83% | 0.93 |
| **Unknown Ethnicity** | 10.61% | 16.75% | < 0.0001 | 10.75% | 10.65% | 0.67 |
| **Asian** | 2.54% | 3.07% | < 0.0001 | 2.57% | 2.51% | 0.62 |
| **Native Hawaiian** | 0.45% | 0.37% | 0.0274 | 0.45% | 0.47% | 0.6618 |
| **American Indian or Alaska Native** | 0.43% | 0.40% | 0.4182 | 0.44% | 0.49% | 0.2996 |
| **BMI Percentile** | 31.2 ± 12.9 | 64.1 ± 31.5 | < 0.0001 | 34.4 ± 17.6 | 49.7 ± 26.2 | < 0.0001 |
| **BMI ≥ 85th Percentile** |  |  | < 0.0001 |  |  | 0.879 |
| **Tobacco use** | 4.93% | 2.82% | < 0.0001 | 5.00% | 5.09% | 0.5657 |

Table A13: Patient Characteristics (BSO + HRT vs No BSO or HRT Radiation SA)

| **Characteristics** |  | **Before matching, No. (%)** | |  | **After matching, No. (%)** | |
| --- | --- | --- | --- | --- | --- | --- |
| **Demographics** | **BSO + HRT** | **Control** | **p-value** | **BSO + HRT** | **Control** | **p-value** |
| **Patients, No.** | 65,396 | 3,237,436 |  | 65,389 | 65,389 |  |
| **Age at Index (Mean +/- SD)** | 59.4 ± 14.6 | 50.6 ± 25.8 | < 0.0001 | 59.4 ± 14.6 | 59.6 ± 14.7 | 0.087 |
| **Not Hispanic or Latino** | 82.45% | 78.96% | < 0.0001 | 82.45% | 82.46% | 0.9768 |
| **White** | 79.34% | 76.51% | < 0.0001 | 79.34% | 79.34% | 0.99 |
| **Unknown Ethnicity** | 10.34% | 16.76% | < 0.0001 | 10.34% | 10.36% | 0.93 |
| **Black or African American** | 10.08% | 11.80% | < 0.0001 | 10.08% | 9.99% | 0.56 |
| **Hispanic or Latino** | 7.21% | 4.29% | < 0.0001 | 7.20% | 7.18% | 0.8893 |
| **Other Race** | 3.61% | 2.79% | < 0.0001 | 3.60% | 3.50% | 0.33 |
| **Unknown Race** | 3.23% | 5.06% | < 0.0001 | 3.23% | 3.20% | 0.75 |
| **Unknown Ethnicity** | 10.34% | 16.76% | < 0.0001 | 10.34% | 10.36% | 0.93 |
| **Asian** | 2.82% | 3.07% | 0.0002 | 2.82% | 2.78% | 0.64 |
| **Native Hawaiian** | 0.45% | 0.37% | 0.0029 | 0.45% | 0.69% | < 0.0001 |
| **American Indian or Alaska Native** | 0.48% | 0.40% | 0.0037 | 0.48% | 0.51% | 0.4764 |
| **BMI Percentile** | 31.1 ± 12.4 | 64.1 ± 31.5 | < 0.0001 | 31.1 ± 12.4 | 52.3 ± 29.2 | < 0.0001 |
| **BMI ≥ 85th Percentile** |  |  | < 0.0001 |  |  | 0.786 |
| **Tobacco use** | 5.16% | 2.82% | < 0.0001 | 5.16% | 5.47% | 0.0107 |

Table A14: Association between BSO and Meningioma Risk Compared with Control

|  | **Risk Difference** | | | | **Risk Ratio** | | **Kaplan-Maier Analysis** | | | | | | | **Cohort Statistics** | | | **Note** |
| --- | --- | --- | --- | --- | --- | --- | --- | --- | --- | --- | --- | --- | --- | --- | --- | --- | --- |
|  | Risk Difference | 95% CI | z | p | Risk Ratio | 95% CI | Survival Probability | Log Rank Test χ² | p-val | Hazard Ratio | 95% CI | Prop χ² | Prop p-val | Patients in Cohort | Patients with Outcome | Risk |  |
| **Any Meningioma (10 yr)** | -0.01% | (-0.049%,0.036%) | -0.287 | 0.7742 | 0.984 | (0.878,1.101) | 98.89% | 0.811 | 0.3677 | 1.054 | (0.94,1.18) | 0.481 | 0.4878 | 158,621 | 594 | 0.37% | BSO + No HRT |
| *D32* |  |  |  |  |  |  | 98.77% |  |  |  |  |  |  | 158,645 | 604 | 0.38% | Control |
| **Intracranial Meningioma (10 yr)** | -0.07% | (-0.107%,-0.036%) | -3.955 | < 0.0001 | 0.759 | (0.662,0.871) | 99.36% | 9.92 | 0.0016 | 0.802 | (0.699,0.92) | 1.077 | 0.2995 | 158,958 | 359 | 0.23% | BSO + No HRT |
| D32.0 |  |  |  |  |  |  | 99.16% |  |  |  |  |  |  | 158,986 | 473 | 0.30% | Control |
| **Spinal Meningioma (10 yr)** | 0.01% | (-0.002%,0.017%) | 1.549 | 0.1213 | 1.5 | (0.895,2.514) | 99.93% | 3.742 | 0.0531 | 1.658 | (0.988,2.783) | 4.49 | 0.0341 | 159,690 | 36 | 0.02% | BSO + No HRT |
| D32.1 |  |  |  |  |  |  | 99.93% |  |  |  |  |  |  | 159,693 | 24 | 0.02% | Control |
| **Any Meningioma (Anytime)** | -0.01% | (-0.053%,0.034%) | -0.427 | 0.6696 | 0.976 | (0.873,1.091) | 97.36% | 0.467 | 0.4945 | 1.04 | (0.929,1.164) | 0.689 | 0.4063 | 158,621 | 605 | 0.38% | BSO + No HRT |
| *D32* |  |  |  |  |  |  | 98.06% |  |  |  |  |  |  | 158,645 | 620 | 0.39% | Control |
| **Intracranial Meningioma (Anytime)** | -0.07% | (-0.108%,-0.036%) | -3.908 | < 0.0001 | 0.764 | (0.667,0.875) | 98.03% | 10.13 | 0.0015 | 0.802 | (0.7,0.919) | 1.325 | 0.2497 | 158,958 | 369 | 0.23% | BSO + No HRT |
| *D32.0* |  |  |  |  |  |  | 98.70% |  |  |  |  |  |  | 158,986 | 483 | 0.30% | Control |
| **Spinal Meningioma (Anytime)** | 0.01% | (-0.003%,0.016%) | 1.27 | 0.204 | 1.385 | (0.836,2.293) | 99.93% | 2.712 | 0.0996 | 1.525 | (0.92,2.529) | 6.338 | 0.0118 | 159,690 | 36 | 0.02% | BSO + No HRT |
| *D32.1* |  |  |  |  |  |  | 99.82% |  |  |  |  |  |  | 159,693 | 26 | 0.02% | Control |

Table A15: Association between HRT and Meningioma Risk Compared with Control

|  | **Risk Difference** | | | | **Risk Ratio** | | **Kaplan-Maier Analysis** | | | | | | | **Cohort Statistics** | | | **Note** |
| --- | --- | --- | --- | --- | --- | --- | --- | --- | --- | --- | --- | --- | --- | --- | --- | --- | --- |
|  | Risk Difference | 95% CI | z | p | Risk Ratio | 95% CI | Survival Probability | Log Rank Test χ² | p-val | Hazard Ratio | 95% CI | Prop χ² | Prop p-val | Patients in Cohort | Patients with Outcome | Risk |  |
| **Any Meningioma (10 yr)** | 0.04% | (0.03%,0.058%) | 6.155 | < 0.0001 | 1.162 | (1.108,1.219) | 99.09% | 0.728 | 0.3936 | 1.021 | (0.973,1.071) | 2.966 | 0.085 | 64,149 | 302 | 0.47% | BSO + HRT |
| *D32* |  |  |  |  |  |  | 99.12% |  |  |  |  |  |  | 64,383 | 191 | 0.30% | Control |
| **Intracranial Meningioma (10 yr)** | 0.03% | (0.018%,0.042%) | 4.972 | < 0.0001 | 1.154 | (1.091,1.221) | 99.37% | 0.315 | 0.5749 | 1.016 | (0.96,1.075) | 8.225 | 0.0041 | 64,345 | 197 | 0.31% | BSO + HRT |
| D32.0 |  |  |  |  |  |  | 99.41% |  |  |  |  |  |  | 64,509 | 151 | 0.23% | Control |
| **Spinal Meningioma (10 yr)** | 0.00% | (-0.004%,0.002%) | -0.458 | 0.6471 | 0.949 | (0.758,1.188) | 99.96% | 2.268 | 0.1321 | 0.841 | (0.672,1.054) | 1.178 | 0.2778 | - | - | - | BSO + HRT |
| D32.1 |  |  |  |  |  |  | 99.96% |  |  |  |  |  |  | - | - | - | Control |
| **Any Meningioma (Anytime)** | 0.05% | (0.032%,0.06%) | 6.376 | < 0.0001 | 1.167 | (1.113,1.223) | 97.87% | 0.922 | 0.337 | 1.024 | (0.976,1.073) | 4.443 | 0.0351 | 64,149 | 307 | 0.48% | BSO + HRT |
| *D32* |  |  |  |  |  |  | 98.57% |  |  |  |  |  |  | 64,383 | 194 | 0.30% | Control |
| **Intracranial Meningioma (Anytime)** | 0.03% | (0.02%,0.044%) | 5.195 | < 0.0001 | 1.16 | (1.097,1.226) | 98.59% | 0.462 | 0.4968 | 1.02 | (0.964,1.078) | 10.32 | 0.0013 | 64,345 | 200 | 0.31% | BSO + HRT |
| *D32.0* |  |  |  |  |  |  | 98.91% |  |  |  |  |  |  | 64,509 | 153 | 0.24% | Control |
| **Spinal Meningioma (Anytime)** | 0.00% | (-0.004%,0.002%) | -0.396 | 0.6922 | 0.956 | (0.765,1.194) | 99.95% | 2.186 | 0.1393 | 0.846 | (0.677,1.056) | 1.802 | 0.1795 | 1,156,200 | 152 | 0.01% | BSO + HRT |
| *D32.1* |  |  |  |  |  |  | 99.94% |  |  |  |  |  |  | 1,156,336 | 159 | 0.01% | Control |

Table A16: Association between BSO + HRT and Meningioma Risk Compared with Control

|  | **Risk Difference** | | | | **Risk Ratio** | | **Kaplan-Maier Analysis** | | | | | | | **Cohort Statistics** | | | **Note** |
| --- | --- | --- | --- | --- | --- | --- | --- | --- | --- | --- | --- | --- | --- | --- | --- | --- | --- |
|  | Risk Difference | 95% CI | z | p | Risk Ratio | 95% CI | Survival Probability | Log Rank Test χ² | p-val | Hazard Ratio | 95% CI | Prop χ² | Prop p-val | Patients in Cohort | Patients with Outcome | Risk |  |
| **Any Meningioma (10 yr)** | 0.17% | (0.107%,0.242%) | 5.049 | < 0.0001 | 1.587 | (1.324,1.902) | 98.66% | 32.183 | < 0.0001 | 1.684 | (1.404,2.021) | 0.149 | 0.6996 | 64,149 | 302 | 0.47% | BSO + HRT |
| *D32* |  |  |  |  |  |  | 99.07% |  |  |  |  |  |  | 64,383 | 191 | 0.30% | Control |
| **Intracranial Meningioma (10 yr)** | 0.07% | (0.015%,0.129%) | 2.493 | 0.0127 | 1.308 | (1.058,1.616) | 99.18% | 8.652 | 0.0033 | 1.375 | (1.111,1.701) | 2.382 | 0.1227 | 64,345 | 197 | 0.31% | BSO + HRT |
| D32.0 |  |  |  |  |  |  | 99.33% |  |  |  |  |  |  | 64,509 | 151 | 0.23% | Control |
| **Spinal Meningioma (10 yr)** | - | - | - | - | - | - | - | - | - | - | - | - | - | - | - | - | BSO + HRT |
| D32.1 |  |  |  |  |  |  | - |  |  |  |  |  |  | - | - | - | Control |
| **Any Meningioma (Anytime)** | 0.18% | (0.109%,0.245%) | 5.099 | < 0.0001 | 1.588 | (1.327,1.9) | 97.56% | 33.637 | < 0.0001 | 1.697 | (1.417,2.034) | 0.634 | 0.426 | 64,149 | 307 | 0.48% | BSO + HRT |
| *D32* |  |  |  |  |  |  | 98.93% |  |  |  |  |  |  | 64,383 | 194 | 0.30% | Control |
| **Intracranial Meningioma (Anytime)** | 0.07% | (0.017%,0.131%) | 2.529 | 0.0114 | 1.311 | (1.062,1.617) | 98.63% | 9.174 | 0.0025 | 1.385 | (1.121,1.711) | 3.57 | 0.0588 | 64,345 | 200 | 0.31% | BSO + HRT |
| *D32.0* |  |  |  |  |  |  | 99.22% |  |  |  |  |  |  | 64,509 | 153 | 0.24% | Control |
| **Spinal Meningioma (Anytime)** | - | - | - | - | - | - | - | - | - | - | - | - | - | - | - | - | BSO + HRT |
| *D32.1* |  |  |  |  |  |  | - |  |  |  |  |  |  | - | - | - | Control |

Table A17: Sensitivity Analysis Incorporating Radiation Therapy in the PSM comparison of BSO + HRT with Control

|  | **Risk Difference** | | | | **Risk Ratio** | | **Kaplan-Maier Analysis** | | | | | | | **Cohort Statistics** | | | **Note** |
| --- | --- | --- | --- | --- | --- | --- | --- | --- | --- | --- | --- | --- | --- | --- | --- | --- | --- |
|  | Risk Difference | 95% CI | z | p | Risk Ratio | 95% CI | Survival Probability | Log Rank Test χ² | p-val | Hazard Ratio | 95% CI | Prop χ² | Prop p-val | Patients in Cohort | Patients with Outcome | Risk |  |
| **Any Meningioma (10 yr)** | 0.16% | (0.095%,0.231%) | 4.682 | < 0.0001 | 1.524 | (1.276,1.82) | 98.65% | 28.699 | < 0.0001 | 1.622 | (1.357,1.94) | 0.289 | 0.591 | 64,694 | 306 | 0.47% | BSO + HRT |
| *D32* |  |  |  |  |  |  | 99.04% |  |  |  |  |  |  | 64,748 | 201 | 0.31% | Control |
| **Intracranial Meningioma (10 yr)** | 0.05% | (-0.004%,0.112%) | 1.83 | 0.0672 | 1.211 | (0.986,1.487) | 99.17% | 5.344 | 0.0208 | 1.275 | (1.037,1.567) | 3.833 | 0.0503 | 64,904 | 201 | 0.31% | BSO + HRT |
| D32.0 |  |  |  |  |  |  | 99.29% |  |  |  |  |  |  | 64,909 | 166 | 0.26% | Control |
| **Spinal Meningioma (10 yr)** | 0.01% | (-0.004%,0.029%) | 1.461 | 0.144 | 1.728 | (0.822,3.63) | 99.86% | 3.441 | 0.0636 | 2.009 | (0.948,4.26) | 0.162 | 0.6871 | 65,359 | 19 | 0.03% | BSO + HRT |
| D32.1 |  |  |  |  |  |  | 99.91% |  |  |  |  |  |  | 65,368 | 11 | 0.02% | Control |
| **Any Meningioma (Anytime)** | 0.16% | (0.095%,0.233%) | 4.685 | < 0.0001 | 1.518 | (1.273,1.811) | 97.54% | 29.4 | < 0.0001 | 1.625 | (1.361,1.94) | 0.572 | 0.4496 | 64,694 | 311 | 0.48% | BSO + HRT |
| *D32* |  |  |  |  |  |  | 98.44% |  |  |  |  |  |  | 64,748 | 205 | 0.32% | Control |
| **Intracranial Meningioma (Anytime)** | 0.05% | (-0.004%,0.112%) | 1.816 | 0.0694 | 1.207 | (0.985,1.48) | 98.62% | 5.407 | 0.0201 | 1.274 | (1.038,1.564) | 4.26 | 0.039 | 64,904 | 204 | 0.31% | BSO + HRT |
| *D32.0* |  |  |  |  |  |  | 98.65% |  |  |  |  |  |  | 64,909 | 169 | 0.26% | Control |
| **Spinal Meningioma (Anytime)** | 0.01% | (-0.004%,0.029%) | 1.461 | 0.144 | 1.728 | (0.822,3.63) | 99.86% | 3.441 | 0.0636 | 2.009 | (0.948,4.26) | 0.069 | 0.7924 | 65,359 | 19 | 0.03% | BSO + HRT |
| *D32.1* |  |  |  |  |  |  | 99.91% |  |  |  |  |  |  | 65,368 | 11 | 0.02% | Control |

Table A18: Sensitivity Analysis of Meningioma Risk after BSO + HRT Using Hysterectomy as a Control Cohort

|  | **Risk Difference** | | | | **Risk Ratio** | | **Kaplan-Maier Analysis** | | | | | | | **Cohort Statistics** | | | **Note** |
| --- | --- | --- | --- | --- | --- | --- | --- | --- | --- | --- | --- | --- | --- | --- | --- | --- | --- |
|  | Risk Difference | 95% CI | z | p | Risk Ratio | 95% CI | Survival Probability | Log Rank Test χ² | p-val | Hazard Ratio | 95% CI | Prop χ² | Prop p-val | Patients in Cohort | Patients with Outcome | Risk |  |
| **Any Meningioma (10 yr)** | 0.15% | (0.075%,0.222%) | 3.977 | < 0.0001 | 1.504 | (1.228,1.841) | 98.76% | 56.013 | < 0.0001 | 2.187 | (1.774,2.696) | 3.475 | 0.0623 | 52,602 | 233 | 0.44% | BSO + HRT |
| *D32* |  |  |  |  |  |  | 99.30% |  |  |  |  |  |  | 52,954 | 156 | 0.30% | Hysterct. |
| **Intracranial Meningioma (10 yr)** | 0.10% | (0.042%,0.157%) | 3.37 | 0.0008 | 1.549 | (1.198,2.002) | 99.27% | 37.122 | < 0.0001 | 2.236 | (1.715,2.914) | 3.02 | 0.0823 | 52,740 | 148 | 0.28% | BSO + HRT |
| D32.0 |  |  |  |  |  |  | 99.57% |  |  |  |  |  |  | 52,992 | 96 | 0.18% | Hysterct. |
| **Spinal Meningioma (10 yr)** | - | - | - | - | - | - | - | - | - | - | - | - | - | - | - | - | BSO + HRT |
| D32.1 |  |  |  |  | - |  | - |  |  |  |  |  |  | - | - | - | Hysterct. |
| **Any Meningioma (Anytime)** | 0.11% | (0.031%,0.183%) | 2.761 | 0.0058 | 1.311 | (1.081,1.59) | 97.73% | 56.896 | < 0.0001 | 2.163 | (1.763,2.654) | 2.878 | 0.0898 | 52,602 | 237 | 0.45% | BSO + HRT |
| *D32* |  |  |  |  |  |  | 96.78% |  |  |  |  |  |  | 52,954 | 182 | 0.34% | Hysterct. |
| **Intracranial Meningioma (Anytime)** | 0.08% | (0.015%,0.135%) | 2.456 | 0.0141 | 1.358 | (1.063,1.735) | 98.87% | 37.344 | < 0.0001 | 2.207 | (1.703,2.86) | 2.51 | 0.1131 | 52,740 | 150 | 0.28% | BSO + HRT |
| *D32.0* |  |  |  |  |  |  | 98.71% |  |  |  |  |  |  | 52,992 | 111 | 0.21% | Hysterct. |
| **Spinal Meningioma (Anytime)** | - | - | - | - | - | - | - | - | - | - | - | - | - | - | - | - | BSO + HRT |
| *D32.1* |  |  |  |  | - |  | - |  |  |  |  |  |  | - | - | - | Hysterct. |

Table A19: Sensitivity Analysis of Meningioma Risk after BSO + HRT Using Cholecystectomy as a Control Cohort

|  | **Risk Difference** | | | | **Risk Ratio** | | **Kaplan-Maier Analysis** | | | | | | | **Cohort Statistics** | | | **Note** |
| --- | --- | --- | --- | --- | --- | --- | --- | --- | --- | --- | --- | --- | --- | --- | --- | --- | --- |
|  | Risk Difference | 95% CI | z | p | Risk Ratio | 95% CI | Survival Probability | Log Rank Test χ² | p-val | Hazard Ratio | 95% CI | Prop χ² | Prop p-val | Patients in Cohort | Patients with Outcome | Risk |  |
| **Any Meningioma (10 yr)** | 0.12% | (0.055%,0.194%) | 3.497 | 0.0005 | 1.356 | (1.142,1.609) | 98.65% | 24.088 | < 0.0001 | 1.538 | (1.293,1.829) | 0.493 | 0.4826 | 64,689 | 306 | 0.47% | BSO + HRT |
| *D32* |  |  |  |  |  |  | 99.10% |  |  |  |  |  |  | 65,073 | 227 | 0.35% | Chole. |
| **Intracranial Meningioma (10 yr)** | 0.09% | (0.034%,0.146%) | 3.171 | 0.0015 | 1.412 | (1.139,1.749) | 99.17% | 18.577 | < 0.0001 | 1.602 | (1.29,1.988) | 1.002 | 0.3169 | 64,899 | 201 | 0.31% | BSO + HRT |
| D32.0 |  |  |  |  |  |  | 99.42% |  |  |  |  |  |  | 65,174 | 143 | 0.22% | Chole. |
| **Spinal Meningioma (10 yr)** | 0.01% | (-0.002%,0.03%) | 1.672 | 0.0945 | 1.9 | (0.884,4.087) | 99.86% | 4.404 | 0.0359 | 2.248 | (1.035,4.884) | 0.425 | 0.5145 | 65,354 | 19 | 0.03% | BSO + HRT |
| D32.1 |  |  |  |  |  |  | 99.96% |  |  |  |  |  |  | 65,371 | ≤ 10* | 0.02% | Chole. |
| **Any Meningioma (Anytime)** | 0.11% | (0.043%,0.184%) | 3.146 | 0.0017 | 1.309 | (1.106,1.549) | 97.54% | 26.105 | < 0.0001 | 1.559 | (1.313,1.851) | 0.168 | 0.6817 | 64,689 | 311 | 0.48% | BSO + HRT |
| *D32* |  |  |  |  |  |  | 65.47% |  |  |  |  |  |  | 65,073 | 239 | 0.37% | Chole. |
| **Intracranial Meningioma (Anytime)** | 0.08% | (0.026%,0.139%) | 2.857 | 0.0043 | 1.357 | (1.1,1.674) | 98.62% | 19.81 | < 0.0001 | 1.62 | (1.307,2.007) | 0.616 | 0.4327 | 64,899 | 204 | 0.31% | BSO + HRT |
| *D32.0* |  |  |  |  |  |  | 98.62% |  |  |  |  |  |  | 65,174 | 151 | 0.23% | Chole. |
| **Spinal Meningioma (Anytime)** | 0.01% | (-0.002%,0.03%) | 1.672 | 0.0945 | 1.9 | (0.884,4.087) | 99.86% | 4.404 | 0.0359 | 2.248 | (1.035,4.884) | 0.403 | 0.5256 | 65,354 | 19 | 0.03% | BSO + HRT |
| *D32.1* |  |  |  |  |  |  | 99.96% |  |  |  |  |  |  | 65,371 | ≤ 10* | 0.02% | Chole. |

Table A20: Likelihood of Surgical Resection Among Patients with Meningioma after BSO + HRT compared with Control

|  | **Risk Difference** | | | | **Risk Ratio** | | **Kaplan-Maier Analysis** | | | | | | | **Cohort Statistics** | | | **Note** |
| --- | --- | --- | --- | --- | --- | --- | --- | --- | --- | --- | --- | --- | --- | --- | --- | --- | --- |
|  | Risk Difference | 95% CI | z | p | Risk Ratio | 95% CI | Survival Probability | Log Rank Test χ² | p-val | Hazard Ratio | 95% CI | Prop χ² | Prop p-val | Patients in Cohort | Patients with Outcome | Risk |  |
| **Meningioma Requiring Resection** | -2.35% | (-3.805%,-0.889%) | -3.112 | 0.0019 | 0.406 | (0.225,0.732) | 97.56% | 8.156 | 0.0043 | 0.429 | (0.236,0.78) | 1.028 | 0.3107 | 935 | 15 | 1.60% | BSO + HRT + MNG |
| *CPT 63276, 63277, 63275, 00QT0ZZ, 00QT3ZZ, 00QT4ZZ, 118886002* |  |  |  |  |  |  | 92.82% |  |  |  |  |  |  | 987 | 39 | 3.95% | MNG Control |
| **Intracranial Meningioma Requiring Resection** | -2.46% | (-3.808%,-1.115%) | -3.528 | 0.0004 | 0.32 | (0.164,0.626) | 98.26% | 11.033 | 0.0009 | 0.335 | (0.17,0.659) | 0.861 | 0.3534 | 948 | 11 | 1.16% | BSO + HRT + MNG |
| 61512, 61519 |  |  |  |  |  |  | 93.62% |  |  |  |  |  |  | 994 | 36 | 3.62% | MNG Control |
| **Spinal Meningioma Requiring Resection** | - | - | - | - | - | - | - | - | - | - | - | - | - | - | - | - | BSO + HRT + MNG |
| 63276, 63277, 63275, 00QT0ZZ, 00QT3ZZ, 00QT4ZZ, 118886002 |  |  |  |  | - |  | - |  |  |  |  |  |  | - | - | - | MNG Control |
| **Meningioma Requiring Radiation** | 0.51% | (-0.696%,1.717%) | 0.833 | 0.4051 | 1.318 | (0.687,2.527) | 95.07% | 1.1 | 0.2942 | 1.42 | (0.735,2.745) | 2.559 | 0.1097 | 944 | 20 | 2.12% | BSO + HRT + MNG |
| *77385, 77386, 1010904, 1010895, D020JZZ, 63620, D02, DG2* |  |  |  |  |  |  | 97.98% |  |  |  |  |  |  | 995 | 16 | 1.61% | MNG Control |

Table A21: Association between BSO + HRT for Fibroids and Meningioma Risk Compared with Control

|  | **Risk Difference** | | | | **Risk Ratio** | | **Kaplan-Maier Analysis** | | | | | | | **Cohort Statistics** | | | **Note** |
| --- | --- | --- | --- | --- | --- | --- | --- | --- | --- | --- | --- | --- | --- | --- | --- | --- | --- |
|  | Risk Difference | 95% CI | z | p | Risk Ratio | 95% CI | Survival Probability | Log Rank Test χ² | p-val | Hazard Ratio | 95% CI | Prop χ² | Prop p-val | Patients in Cohort | Patients with Outcome | Risk |  |
| **Any Meningioma (10 yr)** | 0.40% | (0.232%,0.575%) | 4.611 | < 0.0001 | 2.704 | (1.74,4.202) | 98.53% | 13.244 | 0.0003 | 2.224 | (1.43,3.46) | 0.529 | 0.4672 | 11,394 | 73 | 0.64% | BSO + HRT + Fibroids |
| *D32* |  |  |  |  |  |  | 99.36% |  |  |  |  |  |  | 11,396 | 27 | 0.24% | Control |
| **Intracranial Meningioma (10 yr)** | 0.17% | (0.033%,0.3%) | 2.436 | 0.0149 | 1.905 | (1.124,3.228) | 99.03% | 3.041 | 0.0812 | 1.594 | (0.939,2.705) | 0.822 | 0.3645 | 11,417 | 40 | 0.35% | BSO + HRT + Fibroids |
| D32.0 |  |  |  |  |  |  | 99.49% |  |  |  |  |  |  | 11,416 | 21 | 0.18% | Control |
| **Spinal Meningioma (10 yr)** | - | - | - | - | - | - | - | - | - | - | - | - | - | - | - | - | BSO + HRT + Fibroids |
| D32.1 |  |  |  |  |  |  | - |  |  |  |  |  |  | - | - | - | Control |
| **Any Meningioma (Anytime)** | 0.40% | (0.231%,0.577%) | 4.566 | < 0.0001 | 2.643 | (1.712,4.08) | 97.65% | 12.934 | 0.0003 | 2.18 | (1.41,3.37) | 0.28 | 0.5967 | 11,394 | 74 | 0.65% | BSO + HRT + Fibroids |
| *D32* |  |  |  |  |  |  | 99.01% |  |  |  |  |  |  | 11,396 | 28 | 0.25% | Control |
| **Intracranial Meningioma (Anytime)** | 0.16% | (0.023%,0.293%) | 2.289 | 0.0221 | 1.818 | (1.081,3.057) | 99.03% | 2.629 | 0.105 | 1.534 | (0.911,2.583) | 0.329 | 0.5662 | 11,417 | 40 | 0.35% | BSO + HRT + Fibroids |
| *D32.0* |  |  |  |  |  |  | 99.14% |  |  |  |  |  |  | 11,416 | 22 | 0.19% | Control |
| **Spinal Meningioma (Anytime)** | - | - | - | - | - | - | - | - | - | - | - | - | - | - | - | - | BSO + HRT + Fibroids |
| *D32.1* |  |  |  |  |  |  | - |  |  |  |  |  |  | - | - | - | Control |

Table A22: Association between BSO + HRT for Endometriosis and Meningioma Risk Compared with Control

|  | **Risk Difference** | | | | **Risk Ratio** | | **Kaplan-Maier Analysis** | | | | | | | **Cohort Statistics** | | | **Note** |
| --- | --- | --- | --- | --- | --- | --- | --- | --- | --- | --- | --- | --- | --- | --- | --- | --- | --- |
|  | Risk Difference | 95% CI | z | p | Risk Ratio | 95% CI | Survival Probability | Log Rank Test χ² | p-val | Hazard Ratio | 95% CI | Prop χ² | Prop p-val | Patients in Cohort | Patients with Outcome | Risk |  |
| **Any Meningioma (10 yr)** | 0.32% | (0.145%,0.49%) | 3.602 | 0.0003 | 2.506 | (1.493,4.205) | 98.41% | 8.053 | 0.0045 | 2.087 | (1.241,3.51) | 1.515 | 0 | 9,467 | 50 | 0.53% | BSO + HRT + Endomet. |
| *D32* |  |  |  |  |  |  | 99.39% |  |  |  |  |  |  | 9,488 | 20 | 0.21% | Control |
| **Intracranial Meningioma (10 yr)** | 0.09% | (-0.046%,0.215%) | 1.272 | 0.2035 | 1.503 | (0.799,2.827) | 99.24% | 0.462 | 0.4966 | 1.245 | (0.661,2.346) | 1.663 | 0.1972 | 9,483 | 24 | 0.25% | BSO + HRT + Endomet. |
| D32.0 |  |  |  |  |  |  | 99.58% |  |  |  |  |  |  | 9,499 | 16 | 0.17% | Control |
| **Spinal Meningioma (10 yr)** | - | - | - | - | - | - | - | - | - | - | - | - | - | - | - | - | BSO + HRT + Endomet. |
| D32.1 |  |  |  |  |  |  | - |  |  |  |  |  |  | - | - | - | Control |
| **Any Meningioma (Anytime)** | 0.32% | (0.142%,0.493%) | 3.552 | 0.0004 | 2.434 | (1.465,4.043) | 97.19% | 7.947 | 0.0048 | 2.05 | (1.231,3.413) | 0.966 | 0.3257 | 9,467 | 51 | 0.54% | BSO + HRT + Endomet. |
| *D32* |  |  |  |  |  |  | 99.01% |  |  |  |  |  |  | 9,488 | 21 | 0.22% | Control |
| **Intracranial Meningioma (Anytime)** | 0.07% | (-0.058%,0.206%) | 1.1 | 0.2714 | 1.414 | (0.76,2.631) | 99.24% | 0.306 | 0.58 | 1.192 | (0.639,2.223) | 0.814 | 0.3671 | 9,483 | 24 | 0.25% | BSO + HRT + Endomet. |
| *D32.0* |  |  |  |  |  |  | 99.20% |  |  |  |  |  |  | 9,499 | 17 | 0.18% | Control |
| **Spinal Meningioma (Anytime)** | - | - | - | - | - | - | - | - | - | - | - | - | - | - | - | - | BSO + HRT + Endomet. |
| *D32.1* |  |  |  |  |  |  | - |  |  |  |  |  |  | - | - | - | Control |

Table A23: Association between BSO + HRT for Malignancy and Meningioma Risk Compared with Control

|  | **Risk Difference** | | | | **Risk Ratio** | | **Kaplan-Maier Analysis** | | | | | | | **Cohort Statistics** | | | **Note** |
| --- | --- | --- | --- | --- | --- | --- | --- | --- | --- | --- | --- | --- | --- | --- | --- | --- | --- |
|  | Risk Difference | 95% CI | z | p | Risk Ratio | 95% CI | Survival Probability | Log Rank Test χ² | p-val | Hazard Ratio | 95% CI | Prop χ² | Prop p-val | Patients in Cohort | Patients with Outcome | Risk |  |
| **Any Meningioma (10 yr)** | 0.52% | (0.314%,0.718%) | 5 | < 0.0001 | 2.695 | (1.798,4.04) | 98.42% | 16.375 | < 0.0001 | 2.262 | (1.507,3.395) | 1.681 | 0.1948 | 10,485 | 86 | 0.82% | BSO + HRT + Malign. |
| *D32* |  |  |  |  |  |  | 99.01% |  |  |  |  |  |  | 10,514 | 32 | 0.30% | Control |
| **Intracranial Meningioma (10 yr)** | 0.33% | (0.17%,0.496%) | 4.001 | < 0.0001 | 2.67 | (1.618,4.406) | 99.02% | 10.309 | 0.0013 | 2.227 | (1.348,3.679) | 0.687 | 0.4072 | 10,523 | 56 | 0.53% | BSO + HRT + Malign. |
| D32.0 |  |  |  |  |  |  | 99.38% |  |  |  |  |  |  | 10,536 | 21 | 0.20% | Control |
| **Spinal Meningioma (10 yr)** | - | - | - | - | - | - | - | - | - | - | - | - | - | - | - | - | BSO + HRT + Malign. |
| D32.1 |  |  |  |  |  |  | - |  |  |  |  |  |  | - | - | - | Control |
| **Any Meningioma (Anytime)** | 3.01% | (2.564%,3.459%) | 13.3 | < 0.0001 | 3.517 | (2.884,4.29) |  |  |  |  |  |  |  | 9,910 | 417 | 4.21% | BSO + HRT + Malign. |
| *D32* |  |  |  |  |  |  |  |  |  |  |  |  |  | 10,364 | 124 | 1.20% | Control |
| **Intracranial Meningioma (Anytime)** | 1.07% | (0.845%,1.295%) | 9.303 | < 0.0001 | 7.293 | (4.459,11.928) |  |  |  |  |  |  |  | 10,563 | 131 | 1.24% | BSO + HRT + Malign. |
| *D32.0* |  |  |  |  |  |  |  |  |  |  |  |  |  | 10,585 | 18 | 0.17% | Control |
| **Spinal Meningioma (Anytime)** | 0.51% | (0.303%,0.709%) | 4.887 | < 0.0001 | 2.613 | (1.751,3.9) | 98.42% | 15.79 | < 0.0001 | 2.213 | (1.48,3.309) | 2.1 | 0.1473 | 10,485 | 86 | 0.82% | BSO + HRT + Malign. |
| *D32.1* |  |  |  |  |  |  | 98.57% |  |  |  |  |  |  | 10,514 | 33 | 0.31% | Control |

Table A24: Association between BSO + HRT for Genetic Predisposition to Malignancy and Meningioma Risk Compared with Control

|  | **Risk Difference** | | | | **Risk Ratio** | | **Kaplan-Maier Analysis** | | | | | | | **Cohort Statistics** | | | **Note** |
| --- | --- | --- | --- | --- | --- | --- | --- | --- | --- | --- | --- | --- | --- | --- | --- | --- | --- |
|  | Risk Difference | 95% CI | z | p | Risk Ratio | 95% CI | Survival Probability | Log Rank Test χ² | p-val | Hazard Ratio | 95% CI | Prop χ² | Prop p-val | Patients in Cohort | Patients with Outcome | Risk |  |
| **Any Meningioma (10 yr)** | 0.38% | (0.128%,0.631%) | 2.962 | 0.0031 | 2.543 | (1.34,4.827) | 98.49% | 6.165 | 0.013 | 2.213 | (1.163,4.21) | 0.082 | 0.7744 | 5,277 | 33 | 0.63% | BSO + HRT + Gene. Predispo. |
| *D32* |  |  |  |  |  |  | 99.22% |  |  |  |  |  |  | 5,287 | 13 | 0.25% | Control |
| **Intracranial Meningioma (10 yr)** | 0.19% | (-0.026%,0.405%) | 1.719 | 0.0855 | 1.834 | (0.909,3.703) | 98.83% | 1.754 | 0.1854 | 1.603 | (0.792,3.245) | 0.075 | 0.7836 | 5,292 | 22 | 0.42% | BSO + HRT + Gene. Predispo. |
| D32.0 |  |  |  |  |  |  | 99.27% |  |  |  |  |  |  | 5,295 | 12 | 0.23% | Control |
| **Spinal Meningioma (10 yr)** | - | - | - | - | - | - | - | - | - | - | - | - | - | - | - | - | BSO + HRT + Gene. Predispo. |
| D32.1 |  |  |  |  |  |  | - |  |  |  |  |  |  | - | - | - | Control |
| **Any Meningioma (Anytime)** | 0.38% | (0.123%,0.636%) | 2.9 | 0.0037 | 2.433 | (1.307,4.529) | 92.69% | 6.636 | 0.01 | 2.236 | (1.193,4.191) | 0.251 | 0.6164 | 5,277 | 34 | 0.64% | BSO + HRT + Gene. Predispo. |
| *D32* |  |  |  |  |  |  | 98.57% |  |  |  |  |  |  | 5,287 | 14 | 0.27% | Control |
| **Intracranial Meningioma (Anytime)** | 0.17% | (-0.048%,0.389%) | 1.525 | 0.1271 | 1.693 | (0.854,3.358) | 98.83% | 1.492 | 0.2219 | 1.533 | (0.768,3.058) | 0 | 0.9891 | 5,292 | 22 | 0.42% | BSO + HRT + Gene. Predispo. |
| *D32.0* |  |  |  |  |  |  | 98.61% |  |  |  |  |  |  | 5,295 | 13 | 0.25% | Control |
| **Spinal Meningioma (Anytime)** | - | - | - | - | - | - | - | - | - | - | - | - | - | - | - | - | BSO + HRT + Gene. Predispo. |
| *D32.1* |  |  |  |  |  |  | - |  |  |  |  |  |  | - | - | - | Control |

Table A25: Association between BSO + HRT for ER+/PR+ Breast Cancer and Meningioma Risk Compared with Control

|  | **Risk Difference** | | | | **Risk Ratio** | | **Kaplan-Maier Analysis** | | | | | | | **Cohort Statistics** | | | **Note** |
| --- | --- | --- | --- | --- | --- | --- | --- | --- | --- | --- | --- | --- | --- | --- | --- | --- | --- |
|  | Risk Difference | 95% CI | z | p | Risk Ratio | 95% CI | Survival Probability | Log Rank Test χ² | p-val | Hazard Ratio | 95% CI | Prop χ² | Prop p-val | Patients in Cohort | Patients with Outcome | Risk |  |
| **Any Meningioma (10 yr)** | 0.45% | (0.152%,0.741%) | 2.971 | 0.003 | 2.549 | (1.344,4.837) | 98.71% | 5.201 | 0.0226 | 2.078 | (1.093,3.953) | 0.337 | 0.5618 | 4,493 | 33 | 0.73% | BSO + HRT + ER/PR Breast Ca. |
| *D32* |  |  |  |  |  |  | 99.22% |  |  |  |  |  |  | 4,512 | 13 | 0.29% | Control |
| **Intracranial Meningioma (10 yr)** | - | - | - | - | - | - | - | - | - | - | - | - | - | - | - | - | BSO + HRT + ER/PR Breast Ca. |
| D32.0 |  |  |  |  |  |  | - |  |  |  |  |  |  | - | - | - | Control |
| **Spinal Meningioma (10 yr)** | - | - | - | - | - | - | - | - | - | - | - | - | - | - | - | - | BSO + HRT + ER/PR Breast Ca. |
| D32.1 |  |  |  |  |  |  | - |  |  |  |  |  |  | - | - | - | Control |
| **Any Meningioma (Anytime)** | 0.45% | (0.146%,0.747%) | 2.909 | 0.0036 | 2.439 | (1.311,4.538) | 92.13% | 5.699 | 0.017 | 2.115 | (1.128,3.968) | 0.75 | 0.3864 | 4,493 | 34 | 0.76% | BSO + HRT + ER/PR Breast Ca. |
| *D32* |  |  |  |  |  |  | 98.27% |  |  |  |  |  |  | 4,512 | 14 | 0.31% | Control |
| **Intracranial Meningioma (Anytime)** | 0.27% | (0.022%,0.513%) | 2.138 | 0.0325 | 2.21 | (1.048,4.661) | 92.39% | 2.943 | 0.0863 | 1.921 | (0.9,4.1) | 1.655 | 0.1982 | 4,502 | 22 | 0.49% | BSO + HRT + ER/PR Breast Ca. |
| *D32.0* |  |  |  |  |  |  | 98.59% |  |  |  |  |  |  | 4,522 | ≤ 10* | 0.22% | Control |
| **Spinal Meningioma (Anytime)** | - | - | - | - | - | - | - | - | - | - | - | - | - | - | - | - | BSO + HRT + ER/PR Breast Ca. |
| *D32.1* |  |  |  |  |  |  | - |  |  |  |  |  |  | - | - | - | Control |

Table A26: Association between BSO without HRT for ER+/PR+ Breast Cancer and Meningioma Risk Compared with Control

|  | **Risk Difference** | | | | **Risk Ratio** | | **Kaplan-Maier Analysis** | | | | | | | **Cohort Statistics** | | | **Note** |
| --- | --- | --- | --- | --- | --- | --- | --- | --- | --- | --- | --- | --- | --- | --- | --- | --- | --- |
|  | Risk Difference | 95% CI | z | p | Risk Ratio | 95% CI | Survival Probability | Log Rank Test χ² | p-val | Hazard Ratio | 95% CI | Prop χ² | Prop p-val | Patients in Cohort | Patients with Outcome | Risk |  |
| **Any Meningioma (10 yr)** | -0.05% | (-0.239%,0.14%) | -0.51 | 0.6103 | 0.875 | (0.523,1.464) | 98.71% | 0.098 | 0.7544 | 0.921 | (0.548,1.547) | 0.415 | 0.5194 | 7,830 | 27 | 0.35% | BSO + ER/PR Breast Ca. |
| *D32* |  |  |  |  |  |  | 99.12% |  |  |  |  |  |  | 7,864 | 31 | 0.39% | Control |
| **Intracranial Meningioma (10 yr)** | 0.03% | (-0.135%,0.188%) | 0.32 | 0.7488 | 1.104 | (0.603,2.021) | 99.05% | 0.18 | 0.6716 | 1.14 | (0.621,2.095) | 0.123 | 0.7262 | 7,853 | 22 | 0.28% | BSO + ER/PR Breast Ca. |
| D32.0 |  |  |  |  |  |  | 99.49% |  |  |  |  |  |  | 7,880 | 20 | 0.25% | Control |
| **Spinal Meningioma (10 yr)** | - | - | - | - | - | - | - | - | - | - | - | - | - | - | - | - | BSO + ER/PR Breast Ca. |
| D32.1 |  |  |  |  |  |  | - |  |  |  |  |  |  | - | - | - | Control |
| **Any Meningioma (Anytime)** | 0.09% | (-0.089%,0.266%) | 0.978 | 0.328 | 1.31 | (0.761,2.253) | 49.33% | 2.4 | 0.1214 | 1.548 | (0.887,2.703) | 1.598 | 0.2062 | 8,012 | 30 | 0.37% | BSO + ER/PR Breast Ca. |
| *D32* |  |  |  |  |  |  | 98.57% |  |  |  |  |  |  | 8,045 | 23 | 0.29% | Control |
| **Intracranial Meningioma (Anytime)** | 0.10% | (-0.05%,0.25%) | 1.309 | 0.1904 | 1.538 | (0.803,2.946) | 99.06% | 3.045 | 0.081 | 1.794 | (0.923,3.488) | 0.109 | 0.7408 | 8,036 | 23 | 0.29% | BSO + ER/PR Breast Ca. |
| *D32.0* |  |  |  |  |  |  | 98.83% |  |  |  |  |  |  | 8,062 | 15 | 0.19% | Control |
| **Spinal Meningioma (Anytime)** | - | - | - | - | - | - | - | - | - | - | - | - | - | - | - | - | BSO + ER/PR Breast Ca. |
| *D32.1* |  |  |  |  |  |  | - |  |  |  |  |  |  | - | - | - | Control |

Table A27: Association between BSO without HRT for Non-hormonal Driven or Unspecified Indications and Meningioma Risk Compared with Control

|  | **Risk Difference** | | | | **Risk Ratio** | | **Kaplan-Maier Analysis** | | | | | | | **Cohort Statistics** | | | **Note** |
| --- | --- | --- | --- | --- | --- | --- | --- | --- | --- | --- | --- | --- | --- | --- | --- | --- | --- |
|  | Risk Difference | 95% CI | z | p | Risk Ratio | 95% CI | Survival Probability | Log Rank Test χ² | p-val | Hazard Ratio | 95% CI | Prop χ² | Prop p-val | Patients in Cohort | Patients with Outcome | Risk |  |
| **Any Meningioma (10 yr)** | 0.10% | (0.008%,0.2%) | 2.116 | 0.0343 | 1.268 | (1.017,1.581) | 97.29% | 7.254 | 0.0071 | 1.356 | (1.085,1.693) | 1.358 | 0.2438 | 36,172 | 178 | 0.49% | Other BSO Ind. + BSO + HRT |
| *D32* |  |  |  |  |  |  | 98.22% |  |  |  |  |  |  | 36,335 | 141 | 0.39% | Control |
| **Intracranial Meningioma (10 yr)** | 0.04% | (-0.042%,0.121%) | 0.95 | 0.3421 | 1.133 | (0.875,1.468) | 98.42% | 1.871 | 0.1714 | 1.199 | (0.924,1.555) | 1.735 | 0.1878 | 36,282 | 122 | 0.34% | Other BSO Ind. + BSO + HRT |
| D32.0 |  |  |  |  |  |  | 98.61% |  |  |  |  |  |  | 36,404 | 108 | 0.30% | Control |
| **Spinal Meningioma (10 yr)** |  |  |  |  |  |  |  |  |  |  |  |  |  |  |  |  | Other BSO Ind. + BSO + HRT |
| D32.1 |  |  |  |  |  |  |  |  |  |  |  |  |  |  |  |  | Control) |
| **Any Meningioma (Anytime)** | 0.11% | (0.011%,0.202%) | 2.196 | 0.0281 | 1.283 | (1.027,1.604) | 98.55% | 7.426 | 0.0064 | 1.365 | (1.09,1.709) | 1.453 | 0.2281 | 36,172 | 175 | 0.48% | Other BSO Ind. + BSO + HRT |
| *D32* |  |  |  |  |  |  | 98.84% |  |  |  |  |  |  | 36,335 | 137 | 0.38% | Control) |
| **Intracranial Meningioma (Anytime)** | 0.04% | (-0.038%,0.123%) | 1.027 | 0.3045 | 1.147 | (0.883,1.489) | 99.05% | 1.977 | 0.1597 | 1.207 | (0.928,1.571) | 2.07 | 0.1502 | 36,282 | 120 | 0.33% | Other BSO Ind. + BSO + HRT |
| *D32.0* |  |  |  |  |  |  | 99.18% |  |  |  |  |  |  | 36,404 | 105 | 0.29% | Control |
| **Spinal Meningioma (Anytime)** |  |  |  |  |  |  |  |  |  |  |  |  |  |  |  |  | Other BSO Ind. + BSO + HRT |
| *D32.1* |  |  |  |  |  |  |  |  |  |  |  |  |  |  |  |  | Control |

**Additional Sensitivity Analyses**

Table A28: Patient Characteristics (BSO + HRT vs No BSO or HRT MRI SA)

| **Characteristics** |  | **Before matching, No. (%)** | |  | **After matching, No. (%)** | |
| --- | --- | --- | --- | --- | --- | --- |
| **Demographics** | **BSO + HRT** | **Control** | **p-value** | **BSO + HRT** | **Control** | **p-value** |
| **Patients, No.** | 48,872 | 2,678,027 |  | 48,870 | 48,870 |  |
| **Age at Index (Mean +/- SD)** | 59.4 ± 14.6 | 49.5 ± 26.1 | < 0.0001 | 59.4 ± 14.6 | 59.4 ± 14.6 | 0.9451 |
| **Not Hispanic or Latino** | 83.96% | 80.46% | < 0.0001 | 83.96% | 83.97% | 0.993 |
| **White** | 77.69% | 75.22% | < 0.0001 | 77.69% | 77.69% | 0.99 |
| **Unknown Ethnicity** | 8.37% | 14.58% | < 0.0001 | 8.37% | 8.38% | 0.95 |
| **Black or African American** | 9.94% | 12.86% | < 0.0001 | 9.94% | 9.94% | 1.00 |
| **Hispanic or Latino** | 7.67% | 4.96% | < 0.0001 | 7.66% | 7.65% | 0.9425 |
| **Other Race** | 3.25% | 2.20% | < 0.0001 | 3.25% | 3.25% | 0.99 |
| **Unknown Race** | 4.53% | 5.49% | < 0.0001 | 4.53% | 4.52% | 0.93 |
| **Unknown Ethnicity** | 8.37% | 14.58% | < 0.0001 | 8.37% | 8.38% | 0.95 |
| **Asian** | 3.49% | 3.24% | 0.0019 | 3.49% | 3.50% | 0.96 |
| **Native Hawaiian** | 0.58% | 0.46% | < 0.0001 | 0.58% | 0.59% | 0.83 |
| **American Indian or Alaska Native** | 0.52% | 0.52% | 0.8627 | 0.52% | 0.51% | 0.93 |
| **BMI Percentile** | 71.5 ± 31.2 | 64 ± 31.5 | 0.0686 | 71.5 ± 31.2 | 61.7 ± 35.7 | 0.1229 |
| **BMI ≥ 85th Percentile** |  |  | < 0.0001 |  |  | 0.806 |
| **Tobacco use** | 6.25% | 2.88% | <0.0001 | 6.25% | 6.27% | 0.9054 |
| **MRI Brain w/ Contrast** | 0.295% | 0.082% | <0.0001 | 0.291% | 0.278% | 0.719 |

Table A29: Patient Characteristics (BSO + HRT vs No BSO or HRT with 3+5 Yr Follow-up SA)

| **Characteristics** |  | **Before matching, No. (%)** | |  | **After matching, No. (%)** | |
| --- | --- | --- | --- | --- | --- | --- |
| **Demographics** | **BSO + HRT** | **Control** | **p-value** | **BSO + HRT** | **Control** | **p-value** |
| **Patients, No.** | 48,872 | 2,678,027 |  | 48,872 | 48,872 |  |
| **Age at Index (Mean +/- SD)** | 59.4 ± 14.6 | 49.5 ± 26.1 | < 0.0001 | 59.4 ± 14.6 | 59.4 ± 14.6 | 0.9683 |
| **Not Hispanic or Latino** | 83.96% | 80.46% | < 0.0001 | 83.96% | 83.95% | 0.9652 |
| **White** | 77.69% | 75.22% | < 0.0001 | 77.69% | 77.68% | 0.9877 |
| **Unknown Ethnicity** | 8.37% | 14.58% | < 0.0001 | 8.37% | 8.38% | 0.98 |
| **Black or African American** | 9.94% | 12.86% | < 0.0001 | 9.94% | 9.95% | 0.98 |
| **Hispanic or Latino** | 7.67% | 4.96% | < 0.0001 | 7.67% | 7.67% | 0.9712 |
| **Other Race** | 3.25% | 2.20% | < 0.0001 | 3.25% | 3.26% | 0.99 |
| **Unknown Race** | 4.53% | 5.49% | < 0.0001 | 4.53% | 4.53% | 0.99 |
| **Unknown Ethnicity** | 8.37% | 14.58% | < 0.0001 | 8.37% | 8.38% | 0.98 |
| **Asian** | 3.49% | 3.24% | 0.0019 | 3.49% | 3.49% | 1.00 |
| **Native Hawaiian** | 0.58% | 0.46% | < 0.0001 | 0.58% | 0.58% | 0.97 |
| **American Indian or Alaska Native** | 0.52% | 0.52% | 0.8627 | 0.52% | 0.51% | 0.96 |
| **BMI Percentile** | 71.5 ± 31.2 | 64 ± 31.5 | 0.0686 | 71.5 ± 31.2 | 63 ± 34.8 | 0.1789 |
| **BMI ≥ 85th Percentile** |  |  | < 0.0001 |  |  | 0.806 |
| **Tobacco use** | 6.25% | 2.88% | <0.0001 | 6.25% | 6.25% | 0.9895 |

Table A30: Patient Characteristics (BSO + HRT vs No BSO or HRT Age <52 SA)

| **Characteristics** |  | **Before matching, No. (%)** | |  | **After matching, No. (%)** | |
| --- | --- | --- | --- | --- | --- | --- |
| **Demographics** | **BSO + HRT** | **Control** | **p-value** | **BSO + HRT** | **Control** | **p-value** |
| **Patients, No.** | 16,607 | 1,415,533 |  | 16,607 | 16,607 |  |
| **Age at Index (Mean +/- SD)** | 42.8 ± 7.42 | 24.3 ± 16.6 | < 0.0001 | 42.8 ± 7.42 | 42.8 ± 7.42 | 0.9752 |
| **Not Hispanic or Latino** | 79.58% | 83.41% | < 0.0001 | 79.58% | 79.58% | 1 |
| **White** | 73.93% | 70.99% | < 0.0001 | 73.93% | 73.93% | 0.99 |
| **Unknown Ethnicity** | 10.17% | 8.55% | < 0.0001 | 10.17% | 10.18% | 0.97 |
| **Black or African American** | 11.62% | 13.90% | < 0.0001 | 11.62% | 11.62% | 1 |
| **Hispanic or Latino** | 10.26% | 8.05% | < 0.0001 | 10.26% | 10.24% | 0.9711 |
| **Other Race** | 4.20% | 3.66% | 0.0002 | 4.20% | 4.20% | 1.00 |
| **Unknown Race** | 5.71% | 7.77% | < 0.0001 | 5.71% | 5.70% | 0.98 |
| **Unknown Ethnicity** | 10.17% | 8.55% | < 0.0001 | 10.17% | 10.18% | 0.97 |
| **Asian** | 3.19% | 2.68% | < 0.0001 | 3.19% | 3.19% | 1.00 |
| **Native Hawaiian** | 0.59% | 0.33% | < 0.0001 | 0.59% | 0.60% | 0.89 |
| **American Indian or Alaska Native** | 0.76% | 0.66% | 0.1367 | 0.76% | 0.76% | 1.00 |
| **BMI Percentile** | 71 ± 31.2 | 64 ± 31.5 | 0.0917 | 71 ± 31.2 | 69.4 ± 29.2 | 69.4 ± 29.2 |
| **BMI ≥ 85th Percentile** |  |  | < 0.0001 |  |  | 0.710 |
| **Tobacco use** | 7.78% | 1.92% | <0.0001 | 7.78% | 7.79% | 0.9837 |

Table A31: Patient Characteristics (BSO + HRT vs No BSO or HRT Age >52 SA)

| **Characteristics** |  | **Before matching, No. (%)** | |  | **After matching, No. (%)** | |
| --- | --- | --- | --- | --- | --- | --- |
| **Demographics** | **BSO + HRT** | **Control** | **p-value** | **BSO + HRT** | **Control** | **p-value** |
| **Patients, No.** | 34,800 | 1,537,006 |  | 34,800 | 34,800 |  |
| **Age at Index (Mean +/- SD)** | 66.3 ± 8.96 | 67.5 ± 8.41 | < 0.0001 | 66.3 ± 8.96 | 66.3 ± 8.95 | 0.977 |
| **Not Hispanic or Latino** | 85.11% | 81.10% | < 0.0001 | 85.11% | 85.11% | 0.9915 |
| **White** | 79.26% | 80.51% | < 0.0001 | 79.26% | 79.25% | 0.9851 |
| **Unknown Ethnicity** | 8.58% | 16.56% | < 0.0001 | 8.58% | 8.58% | 0.99 |
| **Black or African American** | 9.52% | 10.68% | < 0.0001 | 9.52% | 9.52% | 0.9897 |
| **Hispanic or Latino** | 6.31% | 2.34% | < 0.0001 | 6.31% | 6.31% | 1 |
| **Other Race** | 2.82% | 1.63% | < 0.0001 | 2.82% | 2.83% | 0.98 |
| **Unknown Race** | 3.97% | 3.08% | < 0.0001 | 3.97% | 3.96% | 0.98 |
| **Unknown Ethnicity** | 8.58% | 16.56% | < 0.0001 | 8.58% | 8.58% | 0.99 |
| **Asian** | 3.47% | 3.24% | 0.0161 | 3.47% | 3.47% | 1.00 |
| **Native Hawaiian** | 0.56% | 0.50% | 0.0865 | 0.56% | 0.56% | 1.00 |
| **American Indian or Alaska Native** | 0.41% | 0.36% | 0.1829 | 0.41% | 0.41% | 0.95 |
| **BMI Percentile** | 100 ± 0 | 92.5 ± 0 | - | 100 ± 0 | - | - |
| **BMI ≥ 85th Percentile** |  |  | 0.6342 |  |  | - |
| **Tobacco use** | 6.01% | 3.84% | <0.0001 | 6.01% | 5.99% | 0.9491 |

Table A32: Patient Characteristics (BSO + HRT vs No BSO or HRT OCP SA)

| **Characteristics** |  | **Before matching, No. (%)** | |  | **After matching, No. (%)** | |
| --- | --- | --- | --- | --- | --- | --- |
| **Demographics** | **BSO + HRT** | **Control** | **p-value** | **BSO + HRT** | **Control** | **p-value** |
| **Patients, No.** | 48,985 | 3,009,067 |  | 48,985 | 48,985 |  |
| **Age at Index (Mean +/- SD)** | 59.3 ± 14.6 | 48.3 ± 26.2 | < 0.0001 | 59.3 ± 14.6 | 59.3 ± 14.6 | 0.9991 |
| **Not Hispanic or Latino** | 83.26% | 82.02% | < 0.0001 | 83.26% | 83.26% | 0.9727 |
| **White** | 78.06% | 76.17% | < 0.0001 | 78.06% | 78.06% | 0.9877 |
| **Unknown Ethnicity** | 9.03% | 13.03% | < 0.0001 | 9.03% | 9.04% | 0.97 |
| **Black or African American** | 9.96% | 11.94% | < 0.0001 | 9.96% | 9.96% | 1 |
| **Hispanic or Latino** | 7.71% | 4.94% | < 0.0001 | 7.71% | 7.71% | 0.9904 |
| **Other Race** | 3.36% | 2.57% | < 0.0001 | 3.36% | 3.36% | 0.99 |
| **Unknown Race** | 4.60% | 5.26% | < 0.0001 | 4.60% | 4.60% | 1.00 |
| **Unknown Ethnicity** | 9.03% | 13.03% | < 0.0001 | 9.03% | 9.04% | 0.97 |
| **Asian** | 3.16% | 3.15% | 0.8874 | 3.16% | 3.16% | 0.99 |
| **Native Hawaiian** | 0.35% | 0.42% | 0.0127 | 0.35% | 0.35% | 0.96 |
| **American Indian or Alaska Native** | 0.52% | 0.49% | 0.3362 | 0.52% | 0.53% | 0.89 |
| **BMI Percentile** | 72.8 ± 29.9 | 64 ± 31.5 | 0.0353 | 72.8 ± 29.9 | 74.9 ± 22.1 | 0.6862 |
| **BMI ≥ 85th Percentile** |  |  | < 0.0001 |  |  | 0.710 |
| **Tobacco use** | 6.20% | 2.60% | <0.0001 | 6.20% | 6.19% | 0.9683 |

Table A33: Sensitivity Analysis Incorporating MRI Brain in the PSM comparison of BSO + HRT with Control

|  | **Risk Difference** | | | | **Risk Ratio** | | **Kaplan-Maier Analysis** | | | | | | | **Cohort Statistics** | | | **Note** |
| --- | --- | --- | --- | --- | --- | --- | --- | --- | --- | --- | --- | --- | --- | --- | --- | --- | --- |
|  | Risk Difference | 95% CI | z | p | Risk Ratio | 95% CI | Survival Probability | Log Rank Test χ² | p-val | Hazard Ratio | 95% CI | Prop χ² | Prop p-val | Patients in Cohort | Patients with Outcome | Risk |  |
| **Any Meningioma (10 yr)** | 0.059% | (-0.023%,0.141%) | 1.418 | 0.1562 | 1.15 | (0.948,1.395) | 98.741% | 4.141 | 0.0419 | 1.223 | (1.007,1.485) | 3.193 | 0.0740 | 48,384 | 220 | 0.455% | Other BSO Ind. + BSO + HRT |
| *D32* |  |  |  |  |  |  | 98.968% |  |  |  |  |  |  | 48,553 | 192 | 0.395% | Control |
| **Intracranial Meningioma (10 yr)** | 0.006% | (-0.016%,0.028%) | 0.54 | 0.5894 | 1.215 | (0.599,2.464) | 99.838% | 0.583 | 0.4452 | 1.318 | (0.647,2.685) | 2.449 | 0.1176 | 48,846 | 17 | 0.035% | Other BSO Ind. + BSO + HRT |
| D32.0 |  |  |  |  |  |  | 99.936% |  |  |  |  |  |  | 48,861 | 14 | 0.029% | Control |
| **Spinal Meningioma (10 yr)** | -0.014% | (-0.081%,0.054%) | -0.394 | 0.6933 | 0.954 | (0.755,1.205) | 99.29% | NaN | 0.9999 | 1 | (0.791,1.264) | 5.836 | 0.0157 | 48,527 | 137 | 0.282% | Other BSO Ind. + BSO + HRT |
| D32.1 |  |  |  |  |  |  | 99.324% |  |  |  |  |  |  | 48,665 | 144 | 0.296% | Control) |
| **Any Meningioma (Anytime)** | 0.139% | (0.056%,0.221%) | 3.306 | 0.0009 | 1.412 | (1.15,1.734) | 97.522% | 14.598 | 0.0001 | 1.492 | (1.214,1.835) | 0.001 | 0.9790 | 45,823 | 218 | 0.476% | Other BSO Ind. + BSO + HRT |
| *D32* |  |  |  |  |  |  | 98.668% |  |  |  |  |  |  | 46,001 | 155 | 0.337% | Control) |
| **Intracranial Meningioma (Anytime)** | - | - | - | - | - | - | - | - | - | - | - | - | - | - | - | - | Other BSO Ind. + BSO + HRT |
| *D32.0* |  |  |  |  |  |  | - |  |  |  |  |  |  | - | - | - | Control |
| **Spinal Meningioma (Anytime)** | 0.023% | (-0.046%,0.091%) | 0.645 | 0.5192 | 1.083 | (0.85,1.381) | 98.824% | 1.081 | 0.2984 | 1.138 | (0.892,1.453) | 0.407 | 0.5236 | 45,950 | 135 | 0.294% | Other BSO Ind. + BSO + HRT |
| *D32.1* |  |  |  |  |  |  |  |  |  |  |  |  |  |  |  |  | Control |

Table A34: Sensitivity Analysis of meningioma risk in patients undergoing BSO + HRT with Control at 3- and 5- year timepoints

|  | **Risk Difference** | | | | **Risk Ratio** | | **Kaplan-Maier Analysis** | | | | | | | **Cohort Statistics** | | | **Note** |
| --- | --- | --- | --- | --- | --- | --- | --- | --- | --- | --- | --- | --- | --- | --- | --- | --- | --- |
|  | Risk Difference | 95% CI | z | p | Risk Ratio | 95% CI | Survival Probability | Log Rank Test χ² | p-val | Hazard Ratio | 95% CI | Prop χ² | Prop p-val | Patients in Cohort | Patients with Outcome | Risk |  |
| **Any Meningioma (10 yr)** | 0.04% | (-0.026%,0.106%) | 1.191 | 0.2335 | 1.156 | (0.91,1.469) | 99.567% | 1.044 | 0.3070 | 1.133 | (0.892,1.44) | 8.18 | 0.0042 | 48,385 | 144 | 0.298% | Other BSO Ind. + BSO + HRT |
| *D32* |  |  |  |  |  |  | 99.645% |  |  |  |  |  |  | 48,570 | 125 | 0.257% | Control |
| **Intracranial Meningioma (10 yr)** | 0% | (-0.018%,0.018%) | 0.001 | 0.9995 | 1 | (0.416,2.403) | 99.971% | 0.003 | 0.9578 | 0.977 | (0.406,2.346) | 3.162 | 0.0754 | 48,848 | ≤ 10* | 0.02% | Other BSO Ind. + BSO + HRT |
| D32.0 |  |  |  |  |  |  | 99.975% |  |  |  |  |  |  | 48,863 | ≤ 10* | 0.02% | Control |
| **Spinal Meningioma (10 yr)** | -0.018% | (-0.073%,0.037%) | -0.634 | 0.5262 | 0.912 | (0.686,1.213) | 99.732% | 0.608 | 0.4357 | 0.893 | (0.671,1.188) | 7.054 | 0.0079 | 48,529 | 90 | 0.185% | Other BSO Ind. + BSO + HRT |
| D32.1 |  |  |  |  |  |  | 99.723% |  |  |  |  |  |  | 48,681 | 99 | 0.203% | Control) |
| **Any Meningioma (Anytime)** | 0.055% | (-0.019%,0.129%) | 1.448 | 0.1477 | 1.17 | (0.946,1.448) | 99.323% | 2.014 | 0.1558 | 1.167 | (0.943,1.444) | 3.296 | 0.0694 | 48,385 | 183 | 0.378% | Other BSO Ind. + BSO + HRT |
| *D32* |  |  |  |  |  |  | 99.456% |  |  |  |  |  |  | 48,570 | 157 | 0.323% | Control) |
| **Intracranial Meningioma (Anytime)** | 0.006% | (-0.014%,0.026%) | 0.601 | 0.5479 | 1.273 | (0.578,2.804) | 99.949% | 0.335 | 0.5627 | 1.262 | (0.573,2.78) | 3.711 | 0.0541 | 48,848 | 14 | 0.029% | Other BSO Ind. + BSO + HRT |
| *D32.0* |  |  |  |  |  |  | 99.97% |  |  |  |  |  |  | 48,863 | 11 | 0.023% | Control |
| **Spinal Meningioma (Anytime)** | -0.022% | (-0.083%,0.04%) | -0.698 | 0.4854 | 0.913 | (0.706,1.18) | 99.607% | 0.547 | 0.4594 | 0.908 | (0.702,1.174) | 2.109 | 0.1464 | 48,529 | 111 | 0.229% | Other BSO Ind. + BSO + HRT |
| *D32.1* |  |  |  |  |  |  |  |  |  |  |  |  |  |  |  |  | Control |

Table A35: Sensitivity Analysis of meningioma risk in patients Age<52 BSO + HRT with Control

|  | **Risk Difference** | | | | **Risk Ratio** | | **Kaplan-Maier Analysis** | | | | | | | **Cohort Statistics** | | | **Note** |
| --- | --- | --- | --- | --- | --- | --- | --- | --- | --- | --- | --- | --- | --- | --- | --- | --- | --- |
|  | Risk Difference | 95% CI | z | p | Risk Ratio | 95% CI | Survival Probability | Log Rank Test χ² | p-val | Hazard Ratio | 95% CI | Prop χ² | Prop p-val | Patients in Cohort | Patients with Outcome | Risk |  |
| **Any Meningioma (10 yr)** | 0.145% | (0.039%,0.251%) | 2.682 | 0.0073 | 1.855 | (1.173,2.936) | 99.097% | 6.744 | 0.0094 | 1.825 | (1.151,2.893) | 0.152 | 0.6962 | 16,537 | 52 | 0.314% | Other BSO Ind. + BSO + HRT |
| *D32* |  |  |  |  |  |  | 99.509% |  |  |  |  |  |  | 16,522 | 28 | 0.169% | Control |
| **Intracranial Meningioma (10 yr)** | - | - | - | - | - | - | - | - | - | - | - | - | - | - | - | - | Other BSO Ind. + BSO + HRT |
| D32.0 |  |  |  |  |  |  | - |  |  |  |  |  |  | - | - | - | Control |
| **Spinal Meningioma (10 yr)** | 0.036% | (-0.049%,0.121%) | 0.829 | 0.4072 | 1.259 | (0.729,2.176) | 99.587% | 0.534 | 0.4648 | 1.226 | (0.709,2.122) | 0.002 | 0.9658 | 16,559 | 29 | 0.175% | Other BSO Ind. + BSO + HRT |
| D32.1 |  |  |  |  |  |  | 99.603% |  |  |  |  |  |  | 16,541 | 23 | 0.139% | Control) |
| **Any Meningioma (Anytime)** | 0.151% | (0.044%,0.258%) | 2.777 | 0.0055 | 1.891 | (1.197,2.988) | 98.804% | 7.433 | 0.0064 | 1.876 | (1.185,2.97) | 0.48 | 0.4886 | 16,537 | 53 | 0.32% | Other BSO Ind. + BSO + HRT |
| *D32* |  |  |  |  |  |  | 99.509% |  |  |  |  |  |  | 16,522 | 28 | 0.169% | Control) |
| **Intracranial Meningioma (Anytime)** | - | - | - | - | - | - | - | - | - | - | - | - | - | - | - | - | Other BSO Ind. + BSO + HRT |
| *D32.0* |  |  |  |  |  |  | - |  |  |  |  |  |  | - | - | - | Control |
| **Spinal Meningioma (Anytime)** | 0.036% | (-0.049%,0.121%) | 0.829 | 0.4072 | 1.259 | (0.729,2.176) | 99.587% | 0.534 | 0.4648 | 1.226 | (0.709,2.122) | 0.003 | 0.9576 | 16,559 | 29 | 0.175% | Other BSO Ind. + BSO + HRT |
| *D32.1* |  |  |  |  |  |  | 99.603% |  |  |  |  |  |  | 16,541 | 23 | 0.139% | Control |

Table A36: Sensitivity Analysis of meningioma risk in patients Age>52 BSO + HRT with Control

|  | **Risk Difference** | | | | **Risk Ratio** | | **Kaplan-Maier Analysis** | | | | | | | **Cohort Statistics** | | | **Note** |
| --- | --- | --- | --- | --- | --- | --- | --- | --- | --- | --- | --- | --- | --- | --- | --- | --- | --- |
|  | Risk Difference | 95% CI | z | p | Risk Ratio | 95% CI | Survival Probability | Log Rank Test χ² | p-val | Hazard Ratio | 95% CI | Prop χ² | Prop p-val | Patients in Cohort | Patients with Outcome | Risk |  |
| **Any Meningioma (10 yr)** | 0.098% | (-0.004%,0.2%) | 1.882 | 0.0598 | 1.233 | (0.991,1.535) | 1.234 | (0.991,1.538) | 98.569% | 6.836 | 0.0089 | 1.34 | (1.075,1.67) | 5.832 | 0.0157 | 34,371 | Other BSO Ind. + BSO + HRT |
| *D32* |  |  |  |  |  |  |  |  | 99.018% |  |  |  |  |  |  | 34,530 | Control |
| **Intracranial Meningioma (10 yr)** | - | - | - | - | - | - | - | - | - | - | - | - | - | - | - | - | Other BSO Ind. + BSO + HRT |
| D32.0 |  |  |  |  |  |  |  |  | - |  |  |  |  |  |  | - | Control |
| **Spinal Meningioma (10 yr)** | 0.039% | (-0.042%,0.119%) | 0.94 | 0.3474 | 1.141 | (0.866,1.503) | 1.141 | (0.866,1.504) | 99.145% | 2.495 | 0.1142 | 1.25 | (0.947,1.648) | 3.82 | 0.0507 | 34,493 | Other BSO Ind. + BSO + HRT |
| D32.1 |  |  |  |  |  |  |  |  | 99.304% |  |  |  |  |  |  | 34,618 | Control) |
| **Any Meningioma (Anytime)** | 0.092% | (-0.011%,0.196%) | 1.745 | 0.0810 | 1.211 | (0.976,1.502) | 1.212 | (0.976,1.505) | 96.212% | 7.102 | 0.0077 | 1.342 | (1.08,1.667) | 5.572 | 0.0183 | 34,371 | Other BSO Ind. + BSO + HRT |
| *D32* |  |  |  |  |  |  |  |  | 98.158% |  |  |  |  |  |  | 34,530 | Control) |
| **Intracranial Meningioma (Anytime)** | - | - | - | - | - | - | - | - | - | - | - | - | - | - | - | - | Other BSO Ind. + BSO + HRT |
| *D32.0* |  |  |  |  |  |  |  |  | - |  |  |  |  |  |  | - | Control |
| **Spinal Meningioma (Anytime)** | 0.033% | (-0.049%,0.115%) | 0.788 | 0.4306 | 1.115 | (0.85,1.462) | 1.116 | (0.85,1.464) | 98.127% | 2.377 | 0.1232 | 1.239 | (0.943,1.628) | 3.308 | 0.0689 | 34,493 | Other BSO Ind. + BSO + HRT |
| *D32.1* |  |  |  |  |  |  |  |  | 98.549% |  |  |  |  |  |  | 34,618 | Control |

Table A37: Sensitivity Analysis of meningioma risk in patients undergoing BSO + HRT (OCP exclusion) with Control

|  | **Risk Difference** | | | | **Risk Ratio** | | **Kaplan-Maier Analysis** | | | | | | | **Cohort Statistics** | | | **Note** |
| --- | --- | --- | --- | --- | --- | --- | --- | --- | --- | --- | --- | --- | --- | --- | --- | --- | --- |
|  | Risk Difference | 95% CI | z | p | Risk Ratio | 95% CI | Survival Probability | Log Rank Test χ² | p-val | Hazard Ratio | 95% CI | Prop χ² | Prop p-val | Patients in Cohort | Patients with Outcome | Risk |  |
| **Any Meningioma (10 yr)** | 0.127% | (0.049%,0.205%) | 3.209 | 0.0013 | 1.399 | (1.138,1.719) | 1.401 | (1.139,1.722) | 98.737% | 13.67 | 0.0002 | 1.474 | (1.199,1.814) | 8.094 | 0.0044 | 48,484 | Other BSO Ind. + BSO + HRT |
| *D32* |  |  |  |  |  |  |  |  | 99.252% |  |  |  |  |  |  | 48,665 | Control |
| **Intracranial Meningioma (10 yr)** | - | - | - | - | - | - | - | - | - | - | - | - | - | - | - | - | Other BSO Ind. + BSO + HRT |
| D32.0 |  |  |  |  |  |  |  |  | - |  |  |  |  |  |  | - | Control |
| **Spinal Meningioma (10 yr)** | 0.054% | (-0.009%,0.117%) | 1.681 | 0.0928 | 1.24 | (0.964,1.593) | 1.24 | (0.964,1.595) | 99.278% | 4.116 | 0.0425 | 1.297 | (1.008,1.67) | 7.944 | 0.0048 | 48,632 | Other BSO Ind. + BSO + HRT |
| D32.1 |  |  |  |  |  |  |  |  | 99.475% |  |  |  |  |  |  | 48,763 | Control) |
| **Any Meningioma (Anytime)** | 0.131% | (0.053%,0.209%) | 3.287 | 0.0010 | 1.407 | (1.147,1.725) | 1.408 | (1.147,1.729) | 97.674% | 14.765 | 0.0001 | 1.493 | (1.215,1.834) | 9.503 | 0.0021 | 48,484 | Other BSO Ind. + BSO + HRT |
| *D32* |  |  |  |  |  |  |  |  | 99.091% |  |  |  |  |  |  | 48,665 | Control) |
| **Intracranial Meningioma (Anytime)** | - | - | - | - | - | - | - | - | - | - | - | - | - | - | - | - | Other BSO Ind. + BSO + HRT |
| *D32.0* |  |  |  |  |  |  |  |  | - |  |  |  |  |  |  | - | Control |
| **Spinal Meningioma (Anytime)** | 0.054% | (-0.009%,0.118%) | 1.668 | 0.0954 | 1.235 | (0.963,1.585) | 1.236 | (0.963,1.587) | 98.807% | 4.287 | 0.0384 | 1.302 | (1.013,1.672) | 8.365 | 0.0038 | 48,632 | Other BSO Ind. + BSO + HRT |
| *D32.1* |  |  |  |  |  |  |  |  | 99.312% |  |  |  |  |  |  | 48,763 | Control |
